# Supplementary material for: Protocol for establishing a child and adolescent twin register for mental health research and capacity building in Sri Lanka and other low and middle-income countries in South Asia
Source: BMJ Open. 2019 Oct 16;9(10):e029332. doi: 10.1136/bmjopen-2019-029332 (PMC6797400; doi:10.1136/bmjopen-2019-029332)
Supplement: Author's manuscript [file bmjopen-2019-029332.draft_revisions.pdf]

BMJ Open

BMJ Open is committed to open peer review. As part of this commitment we make the peer review history of every article we publish publicly available.

When an article is published we post the peer reviewers' comments and the authors' responses online. We also post the versions of the paper that were used during peer review. These are the versions that the peer review comments apply to.

The versions of the paper that follow are the versions that were submitted during the peer review process. They are not the versions of record or the final published versions. They should not be cited or distributed as the published version of this manuscript.

BMJ Open is an open access journal and the full, final, typeset and author-corrected version of record of the manuscript is available on our site with no access controls, subscription charges or pay-per-view fees (http://bmjopen.bmj.com).

If you have any questions on BMJ Open's open peer review process please email info.bmjopen@bmj.com

BMJ Open

Protocol for establishing a child and adolescent twin register for research and capacity building on the aetiology of mental illness in Sri Lanka and other low and middle-income countries in South Asia

Journal:	*BMJ Open*
Manuscript ID	bmjopen-2019-029332
Article Type:	Protocol
Date Submitted by the Author:	20-Mar-2019
Complete List of Authors:	Jayaweera, Kaushalya; Institute for Research & Development, Section of Epidemiology<br>Abeysinghe, Nihal; Institute for Research & Development in Health and Social Care, Sri Lanka, ;<br>De Alwis, Sunil; Ministry of Health, Nutrition and Indigenous Medicine, Medical Services<br>Andras, Alina; Keele University, ;<br>Craig, Jeffrey; Deakin University, School of Medicine<br>Dissanayake, Lasith; Institute for Research and Development, Epidemiology<br>Dziedzic, Krysia; Arthritis Research Campaign National Primary Care Centre<br>Fernando, Buddhika; Research Institute for Primary Care and Health Sciences, ;<br>Glozier, Nick; University of Sydney Brain and Mind Research Institute,<br>Hewamalage, Asiri; Ministry of Health and indegenous Medicine, Sri Lanka, Department of Health;<br>Ives, Jonathan; University of Bristol, Centre for Ethics in Medicine<br>Jordan, Kelvin; Keele University, Primary Care & Health Sciences<br>Kodituwakku, Godwin; Institute for Research & Development in Health and Social Care, Sri Lanka,<br>Mallen, Christian; Keele University, Arthritis Research UK Primary Care Centre<br>Rahman , Omar; Independent University, Public Health<br>Zafar, Shamsa; Air University,<br>Saxena, Alka; Guy's and Saint Thomas' Hospitals NHS Trust, Biomedical Research Centre;<br>Rijsdijk, Fruhling; King's College London, Social, Genetic, and Developmental Psychiatry Centre<br>Saffery, Richard; Murdoch Childrens Research Institute, Cancer and Disease Epigenetics; University of Melbourne, Paediatrics<br>Simonoff, Emily; King's College London, Institute of Psychiatry, Child & Adolescent Psychiatry<br>Yusuf, Rita; Independent University, School of Life Sciences;<br>Zavos, Helena; King's College London, MRC Social, Genetic, and Developmental Psychiatry Centre<br>Sumathipala, Athula; Institute for Research & Development, Research Institute for Primary Care & Health Sciences ; Keele University

	Staffordshire,
Keywords:	Sri Lanka, Twins, Infant child and adolescents, Mental health, Cohort, South Asia, Low and Middle Income Countries (LMIC)

SCHOLARONE™
Manuscripts

Protocol for establishing a child and adolescent twin register for research and capacity building on the aetiology of mental illness in Sri Lanka and other low and middle-income countries in South Asia

Kaushalya Jayaweera[1], Nihal Abeysinghe[1], Sunil De Alwis[2], Alina Andreas[4], Jeffrey Craig[3], Lasith Dissanayake[1], Krysia Dziedzic[4], Buddhika Fernando[1,4], Nicholas Glozier[5], Asiri Hewamalage[2], Jonathan Ives[6], Kelvin Jordan[4], Godwin Kodituwakku[1], Christian Mallen[4], Omar Rahman[7], Shamza Rizwan[8], Alka Saxena[9], Fruhling Rijsdijk[10], Richard Saffery[11], Emily Simonoff[12], Rita Yusuf[7], Helena Zavoz[10], Athula Sumathipala[1,4,12*]. On behalf of SEARCH group

Corresponding Author* - Prof. Athula Sumathipala, Keele University, Staffordshire, ST5 5BG, UK. a.sumathipala@keele.ac.uk, +441782732000

1 Institute for Research and Development, Colombo, Sri Lanka

2 Ministry of Health and Indigenous Medicine, Colombo, Sri Lanka

3 Faculty of Health, Deakin University, Australia

4 Research Institute for Primary Care & Health Sciences, Faculty of Medicine & Health Sciences, Keele University, UK.

5 Brain and Mind Centre, University of Sydney, Australia

6 Center for Ethics and Medicine, University of Bristol, UK

7 Independent University, Bangladesh

8 Health Services Academy, Pakistan

9 NIHR Biomedical Research Centre, Guy's Hospital, Kings College London, UK

10 Social Genetic and Developmental Research Centre, Institute of Psychiatry, Psychology & Neuroscience, King's College London, UK

11 Murdoch Children Research Institute, Victoria, Australia

12 Department of Child and Adolescent Psychiatry, King's College London, Institute of Psychiatry, Psychology & Neuroscience, and NIHR Biomedical Resaerch Centre for Mental Health, UK

Word Count – 5459

For peer review only

ABSTRACT

Introduction:

Worldwide, 10-20% of children and adolescents experience mental disorders, and many continue into adulthood undiagnosed. Half of all mental illnesses begin by age 14 and three-quarters by mid-20s. Little is known about children and adolescents in low and middle-income countries (LMIC), where enormous environmental threats; poverty, war, and conflicts threaten optimal neurodevelopment compared to in high income countries (HIC). Cohort studies provide important information on risks and resilience across the life course, and on childhood, parental and early life environmental characteristics associated with poor health during childhood and in later life. Larger birth cohort studies have been conducted within HIC, but these are not generalizable to LMIC. There are limited longitudinal birth cohort studies in LMIC

Methods:

Our vision is to enhance the impactful research volume in Sri Lanka and South Asia by establishing a centre of excellence for cohort studies. The aim is to establish a register of infant, child and adolescent twins, including mothers pregnant with twins, initially in the districts of Colombo (Western Province) and Vavuniya (Northern Province), with consent from twins or parents to be approached for future research projects. This register will provide the platform to investigate the aetiology of mental illness and the impact of challenges to early brain development in the future. We will expand existing research capacity-building activities on child and adolescent mental health and twin methods, further consolidate existing partnerships and establish new collaborations. The initiative is underpinned by three pillars: developing (1) high quality research, (2) ethics, and (3) patient and public involvement and engagement (PPIE).

Discussion:

This unique register in a LMIC will increase the high-quality research volume, generate a wealth of longitudinal data on mental health and other disciplines, increase awareness and expertise in twin research methods, provide capacity enhancement for junior academics and advances on ethics and PPIE.

MAIN STRENGTHS AND LIMITATIONS SPECIFIC TO THE STUDY

Strengths

- It is a population based register of twins established in a Low and Middle Income Country
- Planning work include data base development, ethics and Public and Patients Involvement and Engagement as three crucial pillars
- It is based on experienced gained through the adult twin register in Colombo Sri Lanka and 15 years of experience

For peer review only

Limitation

- The nature of cohort studies means that only limited paternal data will be available.
- A focus on Sri Lankan twins may limit the generalisability of some findings to other populations.

Keywords

Sri Lanka, Twins, Infant child and adolescents, Mental health, Cohort

BACKGROUND

Gaps in global mental health challenges for child and adolescent mental health in Low and middle-income countries (LMIC)

Worldwide, 10-20% of children and adolescents experience mental disorders, and many transition into adulthood undiagnosed and continue to experience these disorders,[1]. Reasons may include the lack of detection in schools and within families due to associated stigma,[2]. Half of all mental illnesses begin by age 14, and three-quarters by mid-20s, and have significant impact on educational achievement, social relationships, and employability,[3]. WHO-Global Forum, in a survey of mental health research priorities in LMIC including Asia, identified children and adolescents exposed to violence/trauma as prioritized population groups,[4]. Whilst risk and resilience can impact on mental, neurological, and substance use (MNS) disorders and their outcomes; in both cases there is a significant gap in research in LMIC,[5]. Therefore, early stages of life present an important opportunity to prevent mental disorders,[6].

Child and adolescent mental health (CAMH) is especially important in LMIC, as treatment and prevention resources are generally low in comparison to adults,[7]. 'Research and practice in LMIC have focused on children under five years, whereas adolescents are rarely prioritized',[5]. Little is known about the emergence of self-control and regulation in adolescents in LMICs, where enormous environmental threats including poverty, war, and conflicts which threaten optimal neurodevelopment are more common than in HIC,[5].

Children and adolescents exposed to disasters including war are affected by distress, and a variety of psychopathologies,[8,9]. There is also a strong relationship between adolescents exposed to trauma and PTSD or substance abuse,[10–12]. Mental disorders which develop during adolescence can have negative consequences which may result in increased risky decision making in later life,[13,14], substance abuse, depression, anxiety and poor school performance,[3]. Suicide or self-harm is the second leading cause of death among 15-29 years,[15], reflecting high suicide rates in South-East Asia,[16]. However, research on resilience is still scarce in LMIC.

The impact of the challenges to early brain development on mental health and on the aetiology of mental illness

Challenges to early brain development take place from conception. Rapid growth and organ formation occur during the first 12 weeks when the embryo and foetus are most susceptible. Nutrients regulate brain development during the intra-uterine and prenatal

For peer review only

stages of development, and the rapidly developing brain is more vulnerable to nutrient deficiency. A systematic review by Veena and colleagues,[17] concluded that maternal nutritional status during pregnancy influences the offspring's cognitive function during childhood and adolescence. The authors however mention that data is lacking from LMIC and recommended more research from such settings.

Adverse events occurring during the intrauterine or perinatal period are associated with psychosis observed during adulthood,[18].

Neurodevelopmental hypothesis of schizophrenia suggests that, underlying events occurring in the intrauterine or perinatal environment results in an emergence of the psychosis observed during adulthood,[18,19]. Low birth weight and hypoxia are most reliably associated with schizophrenia and gene-environment interaction and gene-environment covariation models are proposed as explanations,[20].

Risk and resilience through a socioecological model in which an adolescent with a particular genome undergoes physical and hormonal transformations within the context of a family, peers, school, work, community and culture, can impact on MNS disorders as an outcome,[5].

Hence, it is crucial to prioritize research on MNS disorders in children and adolescents,[5].

Why Sri Lanka?

Anthropologically, Sri Lanka has a genetically diverse population due to British, Dutch and Portuguese colonisation and Asian immigration. The total population is 20 million with a high literacy rate, and with 33.3% of the population below 18 years of age. It endured a three-decade civil war, and the Asian Tsunami in 2004, resulting in internally displaced people with increased rates of mental disorders,[21]. Eight years post war, it is experiencing demographic transition, rapid globalization, urbanization, and internal/external migration. It has also been proven that children of international labour migrants are at a higher risk of both physical and mental health issues compared to non-migrant families,[22].

Why cohort studies?

Birth cohort studies can provide important information on risks and resilience across the life course. And they can provide data on childhood, parental and early life environmental characteristics associated with poor health during childhood and in later life. Larger longitudinal birth cohort studies have been conducted within HIC, but these are not generalizable to LMIC,[23]. In order to reduce MNS disorders in adolescents in LMIC, Davidson and colleagues,[5] recommended increased support and development of longitudinal cohort studies. Only a limited number of LMIC birth cohort studies exit, and are located in Guatemala, India, Brazil, Philippines,[5]. As findings from these studies cannot be extrapolated to a country such as Sri Lanka due to significant socio-cultural differences further cohorts throughout South Asia are required to generate data to form new strategies to reduce the incidence and impact of MNS disorders in LMIC.

Advantages of twin cohort studies

In longitudinal observational studies, confounding poses a considerable threat to the validity of studies aiming to identify causal mechanisms, creating spurious associations.

For peer review only - http://bmjopen.bmj.com/site/about/guidelines.xhtml

There is also the possibility of unmeasured environmental confounding, occurring when there are one or more contextual factors that affect both the exposure and the outcome. Confounders can be either measured and later be statistically controlled for, or unmeasured. The latter introduces the greatest problems for causal inference. Genetic confounding of identified associations is often a very real possibility but is frequently overlooked by researchers. Twinning is largely unrelated to environmental risk factors such as poverty or social class. Twin samples allow generalizable assessments of associations and the ability to evaluate the extent of both genetic and environmental confounding; one of the reasons for the increased popularity of twin studies over the past decades,[24].

AIMS

1. To establish governance, ethical and logistical systems for the creation this register. To strengthen the ethical framework within the initiative by revising the existing guidelines developed by the IRD.
2. To establish a register of infant, child and adolescent twins, and mothers pregnant with twins in the districts of Colombo (Western Province) and Vavuniya (Northern Province), to be approached for future research projects.
3. Gather preliminary information needed to develop future grant applications.
4. Initiate Patient and Public Involvement and Engagement (PPIE) work in Sri Lanka through the organisation of local events such as workshops and meetings with stake holders.
5. Explore the feasibility of establishing similar twin registers in Bangladesh India, and Pakistan by initiating small scale exploratory studies.
6. Bring together named investigators to from key long-term partner organisations in LMICs and HICs, and establish the planning and coordinating units, and Steering Committee. Research, ethics and Patient Participant Involvement and Engagement (PPIE) expert groups will be developed to strategize and plan relevant activities.

The overall objective is to extend the health and social care research carried out in Sri Lanka, especially by the Institute for Research and Development, Colombo (IRD). Our current twin research follows a 9,500 twin pair cohort of adults,[25] in the population based Sri Lankan Twin Registry,[26]. We intend on expanding to infant, child and adolescent twin studies with the initial aim of identifying research themes and questions which focus on aetiology of mental illness and the impact of challenges to early brain development on mental health, taking a strategic life course approach. The IRD has a strong core team with a variety of expertise and has assembled a team of long term collaborators; local, regional and from HICs who have expertise in the three main pillars of this initiative which are: (i) high quality research, (ii) ethics, and (iii) PPIE. Existing North-South and South-South collaborations will be used to expand ongoing capacity building activities in CAMH, twin methods, genetic research, and ethics.

METHODOLOGY

There is no specific hypothesis driven research to be conducted during the first year of establishing the infant, child and adolescent twin register. However, the broader hypothesis is that using classical twin methods can compare similarity of identical and

non-identical twins to partition the variation of genetic and environmental determinants of disease and traits.

Exact research projects to be undertaken will be decided by methodology experts within the team (Research Experts Group) based on key questions affecting LMIC. Proposals for future research funding applications will be developed during this period of feasibility ascertainment.

The IRD has research instruments for adults, and children and adolescents which have been adapted to both Sinhala and Tamil languages through previous studies, using a more robust method than conventional translation and back translation,[27]. These instruments will be available for future use in projects proposed through this register.

Study Setting; Colombo and Vavuniya districts of Sri Lanka.

The proposed new register will complement and supplement the exiting adult twin register in Colombo district.

Study population

Colombo district is predominantly urban (80%) and has 10% of the Sri Lankan population (2,324,349). The ethnic distribution is 76% Sinhala, 12% Tamil and 11% Moor. 15% of the district population is concentrated in the greater Colombo area where the ethnic distribution is different; 39% Moor, 33% Tamil and 25% Sinhalese. The Vavuniya district has predominantly a rural population (80%) of 172,000, and 83% Tamil, 10% Sinhala and 6% Moor,[28]. These new cohorts provide a good ethnic and urban/rural mix. Vavuniya has been chosen as a comparative setting as it's a more rural region which was affected by the conflict and war.

Inclusion and exclusion

All twins living in Colombo and Vavuniya districts below the age of 18 years will be eligible and have the same opportunity to be registered or not to be registered, based on their, or their parents' or guardians' (for minors) wishes. All mothers pregnant with twins will be eligible to register.

Approaches to identification and recruitment

There will be no recruitment for specific research but only registration in the data base. Twins and their parents, and mothers pregnant with twins will be contacted for consent to be included in the register, and to be approached for future research. Data-base registration will not automatically mean involvement or mandatory participation in research. The recruitment phase will last one year only.

All future projects will be first reviewed and approved by the experts in ethics within the project before proceeding to official ethical review by relevant external ethics review committees.

Twins and their parents will be contacted for consent to be included in the register, and to be approached for future research. Data-base registration does not automatically mean involvement in research or involve mandatory participation in research. To recruit twins and to maximise the outcome of this feasibility study, we decided on 4 source populations: obtaining information through existing twins in the Sri Lankan twin register, maternity hospitals, field visits, and schools.

For peer review only - http://bmjopen.bmj.com/site/about/guidelines.xhtml

Source population A

Twin off-spring are common among dizygotic twins. The adult population based twin register in Colombo comprises 9500 twin pairs of which 4112 twin pairs are of child bearing age. There are also around 1000 pairs from Colombo up to the age 18 voluntarily registered since establishing the adult register. A dedicated team of research assistants will contact these adult twins by phone and postal methods to determine twin off-springs aged up to 18 years and invite them to register. Ethics approval and consent has been obtained previously for contacting these twins.

Source population B

Almost all deliveries take place in major hospitals in Sri Lanka. We will determine the feasibility of using existing hospital records (in Colombo and Vavuniya) from teaching and maternity hospitals to identify new-born twins and mothers attending antenatal clinics pregnant with twins.

Additionally, we will hold discussions with hospital management on feasible variables for data collection, and using anonymised data from the routine health records, assess its completeness and quality. Potential future data to be collected include are detailed in Table 1.

Source Population C

We shall work in collaboration with the Family Health Bureau which is the central organization of the Sri Lankan Ministry of Health responsible for Maternal and Child Health Services. The front-line health worker providing domiciliary care to mothers and children within the community is the Public Health Midwife (PHM). They work within a well demarcated area having a population ranging from about 2000 to 5000 and conduct regular field visits. We will explore the feasibility of identifying twins by PHM to invite for registration.

Source population D

With the support of National Institute for Education and Ministry of Education Sri Lanka, we will conduct a feasibility study of tracing twins through a school survey. There are 584 government schools in both districts and will be possible to identify twins and obtain information about the number of twin pairs in each school, and feasibility of recruitment and engagement. Through the schools we will contact the parents of those twins and provide the information leaflet to register their twin children.

Data to be collected

The primary data collected during the establishment of the register are confined to basic demographic information such as names, dates of birth and contact details. However, information on the possibility of collecting various types of data for future projects will be explored. We will look at the potential data available from school records such as academic performance, entry behaviour assessments administered by Grade 1 upon entry to school. As well as follow-up on their competencies using criteria developed by National Institute of Education, up-to grade 5, and academic attainment through school reports. Additionally, we will also explore the feasibility of obtaining data such as weight trends over the years, immunization status, and any relevant data

For peer review only - http://bmjopen.bmj.com/site/about/guidelines.xhtml

collected by the PHM. With the permission from the hospitals and pregnant mothers we shall also assess the possibility of obtaining recorded data (see Table 1 for details).

*Table 1. **Potential data to be collected in future projects***

**Data from hospitals**	**Data up to the date of delivery**<br>• Expected date of delivery and actual date delivered<br>• Medical and obstetrics related data (maternal complications)<br>• Ultra sound scan data<br>• Method of delivery (normal /instrumental/section)<br>• Neonatal complications<br>• Placentation/zygosity<br>• Birth weight/length/head circumference/Apgar score<br><br>**Post delivery**<br><u>Infant related</u><br>• Placentation/ zygosity<br>• Neonatal complications<br>• Birth weight/ length/head circumference / Apgar score<br>• Weight gain<br>• Mile stones<br>• Breast feeding<br>• Bonding<br><br><u>Maternal</u><br>• Socio demographic data<br>• Type of conception (normal/IVF)<br>• Mood (happiness/sadness)<br>• Maternal nutrition related data<br>• Maternal mental health data<br>• Post-natal complications<br><br><u>Maternal and Paternal</u><br>• Willingness to give consent for bio specimens of the infant
**Data from Primary Health Care workers in the field.**<br><br>**Data will be collected from clinics/house visits.**	**Twin related**<br><u>From health care records available</u><br>• Birth weight/ length/head circumference / Apgar score<br>• Weight trends over the years<br>• Immunization history

For peer review only

	• Health care/illness history including developmental delays<br><br>**Measurements done at home**<br>• Data through questionnaires for:<br>○ Mental health of child<br>○ Cognition of child<br>○ Nutrition related<br><br>**Data from Parents**<br>• Maternal and paternal data as mentioned above<br>• Qualitative interviews from twin's parents:<br>○ including beliefs attitudes and issues about twins<br>○ challenges in rearing twins<br>○ financial implications compared to singletons
**Data from Schools**	**From available school records**<br>• Entry behaviour assessment at Grade 1<br>• Academic attainments indicated through school reports<br>• Records of competency development of children during the primary cycle.<br><br>**Teacher related**<br>• If the twins are in the same school or class<br>• Engagement with peers<br>• Mental health of child

Sample size

Sample size is not relevant as this is a specified research study, however we shall recruit as many twins as possible in both districts. It is also worth noting that twinning rate in Sri Lanka is 10 per 1000 live births,[29]. The Colombo district has 40,000 births per year, with an estimated number of 7,000 twin pairs under the age of 18 years (400X18), and the Vavuniya district 4,000 births per year and 700 twin pairs, up to the age of 18 years. Therefore, any future research will have adequate sample sizes depending on the design.

ETHICAL CONSIDERATIONS

Ethics approval

Ethics approval for this study was obtained from the Ethics Review Committee of Sri Lanka Medical Association of Sri Lanka (ref no: ERC/17-025) and the Keele University's Ethical Review Panel (ref no: ERP3157).

For peer review only

To ensure ethical conduct of the highest standards, by both an internal project governance system and an Ethics Experts Group (EEG) consisting of experts in ethics within this project team, and external procedural ethics review which will seek to achieve an appropriate and justifiable balance between respect for participant autonomy, harm vs benefit, risk and social good.

The key ethical sensitivities arising from future research will focus on participants being a vulnerable group (minors, some having mental illness), involving proxy consent, potential for future complex genetic and omics research, sensitive personal data storage, confidentiality, anonymity and use of and access to data, and international collaborations in a different culture.

Managing the involvement of this vulnerable group

Carrying out research among vulnerable populations needs to be handled with extra caution due to the inherent dilemma in such research. We have taken a proactive approach by setting up the EEG, to provide ethical oversight in all activities, to ensure the right blend of scientific rigor and ethics, while also providing a platform to peer review international collaborative research projects. Protection of twins'/parental interests will be ensured for example by avoiding undue incentives to participate, mechanisms to voice concerns, ensuring awareness of the right to withdraw. The EEG will also set up an internal system of governance to regulate the use of the registry that will work in conform with local ethical review practices.

For all future research project specific ethics review and approval will be obtained from relevant ethics review committees (ERC). We will obtain specific ERC approval and consent from participants for any research involving collection and storage of human material. Data collected will be pseudo anonymised but remain linked to participants using a unique identity number.

The IRD has existing guidelines which have been published,[30], and contain the existing policies and guidelines on access to and sharing data/material. It addresses specific concerns when involving human participants and potential use of human tissue and data collection, documentation, storage and access. These guidelines will be revised and updated accordingly.

In addition, in developing the governance structure we will also incorporate the CIOMS guidelines for future research: more specifically guideline 12; a governance structure in place to ensure the appropriate use of stored data, which is particularly relevant to the proposed work will be considered.

Consent and information provision

Consenting to participate in a cohort study is a long-term commitment, often an uncertain one when specific use of data is not known in advance. Including children and minors in such a project requires parental consent but raises challenges as children grow into mature minors and adults. The EEG will give careful thought to how ongoing consent, assent, and re-consenting will be undertaken as children develop into autonomous persons who can engage in, and make decisions about, research participation. Potential participants will be contacted for consent for register inclusion,

For peer review only - http://bmjopen.bmj.com/site/about/guidelines.xhtml

but data-base registration does not mandate research participation; appropriate consenting processes will be developed to ensure optimal balance between respect for participants' autonomy and overburdening with individual participation requests. Participants will be informed that registration is entirely voluntary, and that there is no obligation for them to take part. Incentives or payments will not be provided for registering in the twin database.

We shall consult the EEG on how best to contact and obtain assesnt/consent from twins and their parents. Information leaflets (which have received ethical approval) will be provided to all potential participants. The leaflets will emphasise the database registration does not involve mandatory participation in research. And that future research will need separate ethics clearance from the country of origin and Sri Lanka.

Data Storage, sharing, access confidentiality and anonymity

Existing IRD institutional policies will be followed to ensure secure data storage and usage to guarantee the obligations of confidentiality and anonymity towards participants. IRD will have ownership of the data collected in future research projects. We will only share anonymised data with academics in the future, however they will not have direct access to the IRD twin data bases. Personal data of participants will be stored in a confidential, password protected databases accessible by only the IRD team. Any documents that contain non-anonymized data will be stored in line with UK Data Protection Act (1998). Consent forms will be kept in a separate location to the research data used for analysis so that no linkage can occur. All datasets and databases will be stored on a secure, password protected computer in IRD's secured data room. Backups of this data will be done regularly and stored along with any hard copy information in a locked filling cabinet of the data room.

For security and confidentiality purposes external access to the register will not be allowed. However, we will share anonymous data for higher degree programmes (in Sri Lanka, Kings College and Keele University, UK); the data sharing policy based on existing institutional policies will be agreed at the outset.

Public involvement

This is a unique opportunity to benefit LMIC, and HIC through pioneering work in PPIE which is a relatively novel concept in Sri Lanka, and South Asia. Evidence from the UK demonstrates that the quality of research is better, and the likelihood of successful recruitment and implementation of the findings is improved when patients and the public are involved in research, [31].

The IRD in collaboration with the Sri Lankan Medical Association and Keele University, UK held a one-day pre-congress workshop at the 131st Anniversary International Medical Congress of the Sri Lanka Medical Association. Resource persons from IRD, Keele University and other guest lectures from Sri Lanka who are experts in PPIE shared their experience and knowledge with 72 workshop participants. This workshop was pre-planned and funded through MRC, UK grant funding obtained for this study.

For peer review only

We aim to establish a culture of PPIE in Sri Lanka, working with both academic colleagues and lay members; in this instance specifically with twins and their parents. Recruiting lay members to a PPIE group to oversee the work described and work with academics to plan and develop future research questions. Training and culturally adapted materials will enhance their engagement. Engaging more professionals/celebrities (parents of twins or twins themselves) in PPIE activities will increase awareness of twin specific issues from a sociological perspective

Testing the feasibility of establishing regional twin registers

We shall also initiate a limited amount of feasibility/exploratory studies with our partner institutions Bangladesh, India, and Pakistan. Feasibility proposals submitted by these institutions will be reviewed by the Steering Committee and experts in ethics within this group, and successful applications will be provided limited seed funding through the Institute of Research & Development, Colombo through allocated funds within the MRC grant for this study. Institute for Mind and Brain was successful in obtaining seed funding to carry out an initial small-scale prevalence study in the local area of Wadakkancherry, Kerala, India in order to estimate the incidence of infant, child and adolescent twins in the local school population. Which was the first step in planning to establish a confidential twin registry in the State of Kerala. The team will also conduct a qualitative study to test the feasibility of creating the Kerala twin registry.

Capacity building

Capacity building is a moral and ethical obligation of externally funded international collaborative research. It will also be bidirectional and collaborative, to advance the local and global agenda is a major component of the project. We have assembled world leading researchers in research methods, ethics, PPIE, and grant development. Ongoing and newly proposed collaborative courses and workshops conducted IRD and this project's collaborators will be open to junior colleagues proposed by the co-investigators. We shall initiate ethics and twin research specific capacity building process in both HIC and LMIC as well, through introductory and advanced courses on twin methods, further strengthening the early and mid-career capacity enhancement by offering the opportunity to work with expert groups. Capacity building benefit specifically on ethics will include enhanced training and knowledge on vulnerable groups (minors, those with mental illness), proxy consent, international collaborations. Funding for such fellowships will be obtained through future grant applications. Capacity building of junior academics across the partnership will develop future research leaders and core skills on child/adolescent mental health and twin research methods.

Dissemination plan

Engagement, communication and dissemination of research and related activities beyond academia are a priority at the IRD. Since its inception, the IRD worked closely with academic partners, policymakers, the public and the electronic/print media. We will promote twin research through electronic media and print, especially using our own trilingual research and science magazine "Gaveshana". Gaveshana is published

For peer review only - http://bmjopen.bmj.com/site/about/guidelines.xhtml

quarterly by the IRD and approved by the Ministry of Education for distribution to schools. Scientific publications in high impact international journals will allow the sharing of knowledge through a wider network. The initiative and the strategy to work beyond twin methods will be widely publicised to attract a wider research community.

TARGETED OUTCOMES OF THE PROJECT AT THE END OF ONE YEAR

Outputs:

1. A sampling frame (the register) of new-born/child/adolescent twins and mothers pregnant with twins with consent to invite for future research.

2. Identification of a core set of potential data variables which are of sufficient quality for research and are feasible to extract from routine records on children's health and education.

3. Development of key research proposals, taking into consideration research gaps in child and adolescent mental health research.

4. Revision of the existing IRD ethics and governance guidelines.

5. PPIE related wok which will include a twin gathering event organized to initiate the process of developing networks and engaging twins in twin research.

DISCUSSSION

The proposed new register will complement and supplement the exiting adult twin register in Colombo district.

This unique register in a LMIC will increase the volume of high-quality research and generate a wealth of longitudinal data on mental health and other disciplines. Phenotypic, biological, and anthropometric data, physical measures and social data will allow the studying of health and wellbeing throughout the life course beyond mental health. It will also take cross cultural research into a global context and will provide a better understanding of the mechanisms and dimensions that underlie vulnerability and resilience to mental illness, as well as the personal, social and economic impact of mental illness as a consequence of a wider ranging external drives and risk factors. It will also popularise twin research methods, provide capacity enhancement for junior academics and advances on ethics and PPIE in Sri Lanka and within the region.

The use of the co-twin control design (using differences within twin pairs to examine the association between a putative environmental risk factor and an outcome variable) is extremely efficient for examining risk factor–outcome associations compared to unmatched designs, and through intervention and non-intervention studies offers many novel utilities for cutting edge research: genomics, genetic approaches for prevention,[32], therapy genetics,[33], and studying genetic determinants of response to pharmacological, as well as psychotherapeutic treatment,[34]. Involving twins in RCTs and randomising concordant MZ twins to two arms can control for many of the potential confounding factors, especially genetic makeup due to matching,[35]. It

For peer review only - http://bmjopen.bmj.com/site/about/guidelines.xhtml

permits large scale epidemiological studies, combining twins with age/gender matched non-twin samples, of both mental disorders and normotypic behaviour.

This initiative provides capacity building in ethics for LMIC and UK researchers, especially on issues related to cutting edge collaborative research in LMIC. Capacity building benefit specifically on ethics will include enhanced training and knowledge on vulnerable groups (minors, those with mental illness), proxy consent, and international collaborations.

Academics in other disciplines will benefit in this endeavour as well. This will be two-fold; firstly, research beyond mental health, and secondly beyond twin research: the collection of a wealth of longitudinal phenotypic, biological, anthropometric and physical measures as well as economic, social and psychological data on families with twins. It also enables pioneering education research.

The initiative has implications for foetal medicine because twins are associated with prematurity, low birth weight, greater intra-uterine growth retardation, more birth defects, birth complications and neonatal mortality.

Social value arising from the project is through the potential impact on many domains. Potential societal impact will come from highlighting the additional economic, social, and psychological impact of being a twin, highlighting twins as a unique societal stratum and as a natural laboratory whose research participation has two-way benefits, and therefore create strong awareness among twins, public and policy planners on the above issues.

Since there is a constellation of exposures that put adolescents at risk of MNS disorders and many of which may be simultaneous outcomes of other exposures, research should, measure multiple factors,[5]. Therefore, the proposed work will offer a significant opportunity for future LMIC research in diverse disciplines.

Acknowledgments

We would like to acknowledge the other members of the **SEARCH Group**: Prof Aasim Ahmad (The Aga Khan University, Pakistan), Dr Achala Jayathilake (Post Graduate Institute of Medicine, Sri Lanka), Prof Ajith Nagahawatte (University of Ruhuna, Sri Lanka), Dr. Anant Bhan (Bioethics and Global Health, India), Dr. Chandima Wickramatilake (University of Ruhuna, Sri Lanka), Dr. Gominda Ponnamperuma (University of Colombo, Sri Lanka), Dr. Lavan Selvarathnam (Ministry of Health, Sri Lanka), Prof Preethi Udagama (University of Colombo, Sri Lanka), Dr. Sorcha Uí Chonnachtaigh, Dr. Steven Blackburn, Dr. Tom Shepherd, and Dr. Toby Helliwell (from (Keele University, UK). Dr. Sudath Samaraweera (Ministry of Health, Sri Lanka), Dr. Thamasi Makuloluwa (General Sir John Kotelawala Defence University, Sri Lanka), Dr. Yatan Balhara (All India Institute of Medical Sciences, Delhi), and Ms Ruwini Cooray (Institute for Research & Development).

We would like to acknowledge Dr. Jayanthi Gunesekara (National Institute of Education), Mrs. Jayani Tillakaratne (Ministry of Education), Mr. Saman Indrajith (The

For peer review only - http://bmjopen.bmj.com/site/about/guidelines.xhtml

Island Newspaper, Sri Lanka). We also highly appreciate the ongoing support from Dr. Nethanjalee Mapitigama, Dr Kaushalya Kasthuriarachchi, and Dr. Hiranya Jayawickrama of the Family Health Bureau, and Dr. Ruwan Wijayamuni, Chief Medical Officer, Colombo Municipal Council. We would also like to thank higher officials of the Ministry of Health and the Ministry of Education.

We gratefully acknowledge members of the Institute for Research & Development, Dr. Irena Zwierska and Ms. Rebecca Parker at Keele University who have contributed immensely to the preparation and execution of the project.

Funding

This study is supported by a one year pump priming grant awarded by the Medical Research Council, UK (Grant number: MC_PC_MR/R018448/1). AS currently receives salary support in kind from the Midland Partnership Foundation NHS Trust, UK.

Authors' contributions

All authors contributed to the drafting of original funding application which is the basis of this protocol paper. KJ and AS contributed at all stages of planning and writing the article based on that application. All authors contributed to different drafts of the paper and finally approved the manuscript for submission.

Competing interests

The authors declare that they have no competing interests.

References:

1 Patel V, Flisher AJ, Hetrick S, *et al.* Mental health of young people: a global public-health challenge. *Lancet* 2007;**369**:1302–13. doi:10.1016/S0140-6736(07)60368-7

2 Kieling C, Baker-Henningham H, Belfer MM, *et al.* Child and adolescent mental health worldwide: evidence for action. *Lancet (London, England)* 2011;**378**:1515–25. doi:10.1016/S0140-6736(11)60827-1

3 Ranasinghe S, Ramesh S, Jacobsen KH. Hygiene and mental health among middle school students in India and 11 other countries. *J Infect Public Health* 2016;**9**:429–35. doi:10.1016/j.jiph.2015.11.007

4 Sharan P, Gallo C, Gureje O, *et al.* Mental health research priorities in low- and middle-income countries of Africa, Asia, Latin America and the Caribbean. *Br J Psychiatry* 2009;**195**:354–63. doi:10.1192/bjp.bp.108.050187

For peer review only - http://bmjopen.bmj.com/site/about/guidelines.xhtml

5 Davidson LL, Grigorenko EL, Boivin MJ, *et al.* A focus on adolescence to reduce neurological, mental health and substance-use disability. *Nature* 2015;**527**:S161–6. doi:10.1038/nature16030

6 WHO. Mental Health Action Plan 2013-2020. *WHO Libr Cat DataLibrary Cat Data* 2013;:1–44. doi:ISBN 978 92 4 150602 1

7 World Health Organization. Atlas: Child and Adolescent Mental Health Resources: Global Concerns, Implications for the Future. 2005. doi:10.3109/08039487509097356

8 et al. Jong, Joop; Freidman, Matthew; Green B. *Trauma Interventions in War and Peace Prevention, Practice, and Policy*. 2004.

9 Betancourt TS, Khan KT. The mental health of children affected by armed conflict: protective processes and pathways to resilience. *Int Rev Psychiatry* 2008;**20**:317–28. doi:10.1080/09540260802090363

10 Deykin EY, Buka SL. Prevalence and risk factors for posttraumatic stress disorder among chemically dependent adolescents. *Am J Psychiatry* 1997;**154**:752–7. doi:10.1176/ajp.154.6.752

11 Perkonigg A, Kessler RC, Storz S, *et al.* Traumatic events and post-traumatic stress disorder in the community: prevalence, risk factors and comorbidity. *Acta Psychiatr Scand* 2000;**101**:46–59. doi:10.1034/j.1600-0447.2000.101001046.x

12 Giaconia RM, Reinherz HZ, Hauf AA, *et al.* Comorbidity of Substance Use and Post-Traumatic Stress Disorders in a Community Sample of Adolescents. *Am J Orthopsychiatry* 2000;**70**:253–62. doi:10.1037/ h0087634

13 Hallfors DD, Waller MW, Ford CA, *et al.* Adolescent depression and suicide risk: Association with sex and drug behavior. *Am J Prev Med* 2004;**27**:224–31. doi:10.1016/j.amepre.2004.06.001

14 Shrier LA, Harris SK, Sternberg M, *et al.* Associations of depression, self-esteem, and substance use with sexual risk among adolescents. *Prev Med (Baltim)* 2001;**33**:179–89. doi:10.1006/pmed.2001.0869

15 World Health Organization. WORLD HEALTH STATISTICS - MONITORING HEALTH FOR THE SDGs. *World Heal Organ* 2016;:1.121. doi:10.1017/CBO9781107415324.004

16 World Health Organization. *Mental Health Status of Adolescents in South-East Asia : Evidence for Action*. 2017.

17 Veena SR, Gale CR, Krishnaveni G V, *et al.* Association between maternal nutritional status in pregnancy and offspring cognitive function during childhood and adolescence; a systematic review. *BMC Pregnancy Childbirth* 2016;**16**:220. doi:10.1186/s12884-016-1011-z

18 Jenkins TA. Perinatal complications and schizophrenia: Involvement of the immune system. Front. Neurosci. 2013;**7**:110. doi:10.3389/fnins.2013.00110

For peer review only

19 Rasmussen B, Hendrieckx C, Clarke B, *et al.* Psychosocial issues of women with type 1 diabetes transitioning to motherhood: A structured literature review. *BMC Pregnancy Childbirth* 2013;**13**. doi:10.1186/1471-2393-13-218

20 Forsyth JK, Ellman LM, Tanskanen A, *et al.* Genetic risk for schizophrenia, obstetric complications, and adolescent school outcome: Evidence for gene-environment interaction. *Schizophr Bull* 2013;**39**:1067–76. doi:10.1093/schbul/sbs098

21 Siriwardhana C. Mental health of displaced and returnee populations: Insight from the Sri Lankan post-conflict experience. *Confl Health* 2015;**9**:22. doi:10.1186/s13031-015-0049-2

22 Wickramage K, Siriwardhana C, Vidanapathirana P, *et al.* Risk of mental health and nutritional problems for left-behind children of international labor migrants. *BMC Psychiatry* 2015;**15**:39. doi:10.1186/s12888-015-0412-2

23 Baxter AJ, Patton G, Scott KM, *et al.* Global Epidemiology of Mental Disorders: What Are We Missing? *PLoS One* 2013;**8**:e65514. doi:10.1371/journal.pone.0065514

24 Gjerde LC, Czajkowski N, Roysamb E, *et al.* A longitudinal, population-based twin study of avoidant and obsessive-compulsive personality disorder traits from early to middle adulthood. *Psychol Med* 2015;**45**:3539–48. doi:10.1017/S0033291715001440

25 Jayaweera K, Aschan L, Pannala G, *et al.* The Colombo Twin and Singleton Follow-up Study: a population based twin study of psychiatric disorders and metabolic syndrome in Sri Lanka. *BMC Public Health* 2018;**18**:145.http://gateway.proquest.com/openurl?ctx_ver=Z39.88-2004&res_id=xri:pqm&req_dat=xri:pqil:pq_clntid=49126&rft_val_fmt=ori/fmt:kev:mtx:journal&genre=article&issn=1471-2458&volume=18&issue=1&spage=145

26 Siribaddana SH, Siriwardane WD, Hewage SN, *et al.* Update from Sri Lankan Twin Registry: Establishment of a population-based twin register and ongoing project on common mental disorders, alcohol abuse and suicidal ideations. Twin Res. Hum. Genet. 2006;**9**:868–74. doi:10.1375/183242706779462679

27 Sumathipala A, Murray J. New approach to translating instruments for cross-cultural research: A combined qualitative and quantitative approach for translation and consensus generation. *Int J Methods Psychiatr Res* 2000;**9**:87–95. doi:10.1002/mpr.83

28 Census of Population and Housing 2012 Department of Census & Statistics and Ministry of Policy Planning and Economic Affairs. 2015. http://www.statistics.gov.lk/PopHouSat/CPH2011/Pages/Activities/Reports/FinalReport/FinalReportE.pdf (accessed 24 Oct 2018).

29 Medical Statistics Unit. Annual Health Statistics 2015. 2017.

30 Sumathipala A, Siribaddana S. *Research Ethics from a Developing World Perspective*. Vijitha Yapa publishers, Colombo 2003.

For peer review only

https://search.informit.com.au/fullText;dn=978230397803004;res=E-LIBRARY (accessed 14 Aug 2017).

31 Mockford C, Staniszewska S, Griffiths F, *et al.* The impact of patient and public involvement on UK NHS health care: A systematic review. Int. J. Qual. Heal. Care. 2012;**24**:28–38. doi:10.1093/intqhc/mzr066

32 WHO. Community genetic services: report of a WHO consultation on community genetics in low- and middle-income countries. 2010. doi:10.1159/000080789

33 Plomin R, Haworth CM a. Genetics and Intervention research. *Perspect Psychol Sci* 2010;**5**:557–63. doi:10.1177/1745691610383513

34 Eley TC, Hudson JL, Creswell C, *et al.* Therapygenetics: The 5HTTLPR and response to psychological therapy. *Mol Psychiatry* 2012;**17**:236–7. doi:10.1038/mp.2011.132

35 Sumathipala A, Yelland L, Green D, *et al.* Twins as participants in randomised controlled trials: a review of published literature. *Twin Res Hum Genet*

For peer review only

BMJ Open

Protocol for establishing a child and adolescent twin register for research and capacity building on the aetiology of mental illness in Sri Lanka and other low and middle-income countries in South Asia

Journal:	*BMJ Open*
Manuscript ID	bmjopen-2019-029332.R1
Article Type:	Protocol
Date Submitted by the Author:	11-Jun-2019

Complete List of Authors:	Jayaweera, Kaushalya; Institute for Research & Development, Section of Epidemiology<br>Abeysinghe, Nihal; Institute for Research & Development in Health and Social Care, Sri Lanka, ;<br>De Alwis, Sunil; Ministry of Health, Nutrition and Indigenous Medicine, Medical Services<br>Andras, Alina; Keele University, ;<br>Craig, Jeffrey; Deakin University, School of Medicine<br>Dissanayake, Lasith; Institute for Research and Development, Epidemiology<br>Dziedzic, Krysia; Arthritis Research Campaign National Primary Care Centre<br>Fernando, Buddhika; Research Institute for Primary Care and Health Sciences, ;<br>Glozier, Nick; University of Sydney Brain and Mind Research Institute,<br>Hewamalage, Asiri; Ministry of Health and indegenous Medicine, Sri Lanka, Department of Health;<br>Ives, Jonathan; University of Bristol, Centre for Ethics in Medicine<br>Jordan, Kelvin; Keele University, Primary Care & Health Sciences<br>Kodituwakku, Godwin; Institute for Research & Development in Health and Social Care, Sri Lanka,<br>Mallen, Christian; Keele University, Arthritis Research UK Primary Care Centre<br>Rahman , Omar; Independent University, Public Health<br>Zafar, Shamsa; Air University,<br>Saxena, Alka; Guy's and Saint Thomas' Hospitals NHS Trust, Biomedical Research Centre;<br>Rijsdijk, Fruhling; King's College London, Social, Genetic, and Developmental Psychiatry Centre<br>Saffery, Richard; Murdoch Childrens Research Institute, Cancer and Disease Epigenetics; University of Melbourne, Paediatrics<br>Simonoff, Emily; King's College London, Institute of Psychiatry, Child & Adolescent Psychiatry<br>Yusuf, Rita; Independent University, School of Life Sciences;<br>Zavos, Helena; King's College London, MRC Social, Genetic, and Developmental Psychiatry Centre<br>Sumathipala, Athula; Institute for Research & Development, Research Institute for Primary Care & Health Sciences ; Keele University

	Staffordshire,
**Primary Subject Heading**:	Epidemiology
Secondary Subject Heading:	Genetics and genomics, Patient-centred medicine, Global health
Keywords:	Sri Lanka, Twins, Infant child and adolescents, Mental health, Cohort, South Asia, Low and Middle Income Countries (LMIC)

SCHOLARONE™ Manuscripts

Protocol for establishing a child and adolescent twin register for research and capacity building on the aetiology of mental illness in Sri Lanka and other low and middle-income countries in South Asia

Kaushalya Jayaweera[1], Nihal Abeysinghe[1], Sunil De Alwis[2], Alina Andras[4], Jeffrey Craig[3], Lasith Dissanayake[1], Krysia Dziedzic[4], Buddhika Fernando[1,4], Nick Glozier[5], Asiri Hewamalage[2], Jonathan Ives[6], Kelvin Jordan[4], Godwin Kodituwakku[1], Christian Mallen[4], Omar Rahman[7], Shamsa Zafar[8], Alka Saxena[9], Fruhling Rijsdijk[10], Richard Saffery[11], Emily Simonoff[12], Rita Yusuf[7], Helena Zavos[10], Athula Sumathipala[1,4,12*]. On behalf of SEARCH group

Corresponding Author* - Prof. Athula Sumathipala, Keele University, Staffordshire, ST5 5BG, UK. a.sumathipala@keele.ac.uk, +441782732000

1 Institute for Research and Development in Health & Social Care, Colombo, Sri Lanka

2 Ministry of Health and Indigenous Medicine, Colombo, Sri Lanka

3 Faculty of Health, Deakin University, Australia

4 Research Institute for Primary Care & Health Sciences, Faculty of Medicine & Health Sciences, Keele University, UK.

5 Brain and Mind Centre, University of Sydney, Australia

6 Center for Ethics and Medicine, University of Bristol, UK

7 Independent University, Bangladesh

8 Health Services Academy, Pakistan

9 NIHR Biomedical Research Centre, Guy's Hospital, Kings College London, UK

10 Social Genetic and Developmental Research Centre, Institute of Psychiatry, Psychology & Neuroscience, King's College London, UK

11 Murdoch Children Research Institute, Victoria, Australia

12 Department of Child and Adolescent Psychiatry, King's College London, Institute of Psychiatry, Psychology & Neuroscience, and NIHR Biomedical Research Centre for Mental Health, UK

Word Count – 5759

For peer review only

ABSTRACT

Introduction:

Worldwide, 10-20% of children and adolescents experience mental disorders, and many continue into adulthood undiagnosed. Half of all mental illnesses begin by age 14 and three-quarters by mid-20s. Little is known about children and adolescents in low and middle-income countries (LMIC), where enormous environmental threats; poverty, war, and conflicts threaten optimal neurodevelopment compared to in high income countries (HIC). Cohort studies provide important information on risks and resilience across the life course, and on childhood, parental and early life environmental characteristics associated with poor health during childhood and in later life. Larger birth cohort studies have been conducted within HIC, but these are not generalizable to LMIC. There are limited longitudinal birth cohort studies in LMIC.

Methods:

Our vision is to enhance the impactful research volume in Sri Lanka and South Asia by establishing a centre of excellence for cohort studies. The aim is to establish a register of infant, child and adolescent twins, including mothers pregnant with twins, initially in the districts of Colombo (Western Province) and Vavuniya (Northern Province), with consent from twins or parents to be approached for future research projects. This register will provide the platform to investigate the aetiology of mental illness and the impact of challenges to early brain development in the future. We will expand existing research capacity-building activities on child and adolescent mental health and twin methods, further consolidate existing partnerships and establish new collaborations. The initiative is underpinned by three pillars: developing (1) high quality research, (2) ethics, and (3) patient and public involvement and engagement (PPIE).

Discussion:

This unique register in a LMIC will increase the high-quality research volume, generate a wealth of longitudinal data on mental health and other disciplines, increase awareness and expertise in twin research methods, provide capacity enhancement for junior academics and advances on ethics and PPIE.

MAIN STRENGTHS AND LIMITATIONS SPECIFIC TO THE STUDY

Strengths

- It is a population-based register of twins established in a Low- and Middle-Income Country
- Planning work include data base development, ethics and Public and Patients Involvement and Engagement as three crucial pillars

For peer review only

- It is based on experienced gained through the adult twin register in Colombo Sri Lanka and 15 years of experience

Limitation

- The nature of cohort studies means that only limited paternal data will be available.
- A focus on Sri Lankan twins may limit the generalisability of some findings to other populations.

Keywords

Sri Lanka, Twins, Infant child and adolescents, Mental health, Cohort

BACKGROUND

Gaps in global mental health challenges for child and adolescent mental health in Low and middle-income countries (LMIC)

The multifactorial aetiology of mental health disorder in the developmental age

Challenges to early brain development take place in intra uterine life and continue throughout childhood and adolescence. Nutrients and growth factors regulate brain development during foetal and early postnatal life and rapidly developing brain is more vulnerable to nutrient insufficiency,[1]. Low birth weight (LBW) and hypoxia are among the environmental factors most reliably associated with schizophrenia. Poverty and adversities such as mother's ill health and high stress during pregnancy; early loss of parents via death or abandonment; witnessing inter-parental violence; dysfunctional parenting (particularly 'affectionless over control'); parental substance misuse, mental health problems and criminal behaviour; childhood sexual, physical and emotional abuse; childhood emotional or physical neglect; bullying; childhood medical illness; and war trauma have also been found to influence outcome,[2,3].

The consequences of disasters are well known causes of a variety of psychological and psychiatric disorders,[4]. Children and adolescents exposed to war related traumatic events are affected mental health distress, and in certain cases long term psychopathology,[5]. There is also a strong relationship between adolescents exposed to trauma and suffering from PTSD and substance abuse. Studies have shown that more than half of the young people with PTSD subsequently develop substance abuse problems,[6].

Prolonged adolescent major depressive disorder left untreated may reoccur in later adult life,[7]. However, diagnosis and treatment of depression during adolescence is beneficial in reducing adult depression symptoms,[8].

Mental health disorders which develop during adolescence can have negative consequences which may result in increased risky decision making in later life,[9,10].

For peer review only - http://bmjopen.bmj.com/site/about/guidelines.xhtml

Social support is essential to young people for coping and protecting against the negative effects of stressors and for the promotion of positive mental health outcomes,[11,12]. Research findings indicate an inverse relationship between social support and depression,[12,13].

Some of these adversities have been shown to be intergenerational, so that parents who themselves suffered in childhood struggle to provide an optimum environment for their own children. It is very likely that these types of events have their impact in interaction with other factors such as genetic predisposition and epigenetic processes,[3]. Both gene-environment interaction and gene-environment covariation models have been proposed as explanations,[14].

Hence, there is a crucial need to prioritize global health research directed at neurological, mental health and substance-use disorders in adolescents,[15] and there is a need as well as potential for preventive actions on these environmental factors.

Worldwide, 10-20% of children and adolescents experience mental disorders, and many transition into adulthood undiagnosed and continue to experience these disorders,[16]. Reasons may include, lack of detection in schools and within families due to associated stigma,[17] inadequate social support which has an inverse relationship with depression and is essential to young people for coping,[12] children and adolescents being exposed to violence/trauma,[18]. Looking from a socioecological model in which an adolescent with a particular genome undergoes physical and hormonal transformations within the context of a family, peers, school, work, community and culture all of which can be instrumental in determining adolescent NMS outcomes,[15] even though faced high levels of mental health-related challenges, treatment and prevention resources for CAMH are generally low, even compared to adults,[19]. Half of all mental illnesses begin by age 14, and three-quarters by mid-20s, and have significant impact on educational achievement, social relationships, well-being and employability,[20]. WHO-Global Forum, in a survey of mental health research priorities in LMIC including Asia, identified children and adolescents exposed to violence/trauma as prioritized population groups,[18]. Whilst risk and resilience can impact on mental, neurological, and substance use (MNS) disorders and their outcomes; in both cases there is a significant gap in research in LMIC,[15]. Therefore, early stages of life present an important opportunity to prevent mental disorders,[21].

Child and adolescent mental health (CAMH) is especially important in LMIC, as treatment and prevention resources are generally low in comparison to adults,[19]. 'Research and practice in LMIC have focused on children under five years, whereas adolescents are rarely prioritized',[15]. Little is known about the emergence of self-control and regulation in adolescents in LMICs, where enormous environmental threats including poverty, war, and conflicts which threaten optimal neurodevelopment are more common than in high income countries (HIC),[15].

For peer review only - http://bmjopen.bmj.com/site/about/guidelines.xhtml

Adolescence is a critical developmental psychological period. Mental health is a key factor in the overall health and wellbeing of a child. Prolonged adolescent major depressive disorder left untreated may reoccur in later adult life,[7]. Children and adolescents exposed to disasters including war are affected by distress, and a variety of psychopathologies,[4,5]. There is also a strong relationship between adolescents exposed to trauma and PTSD or substance abuse,[6,22,23]. Mental disorders which develop during adolescence can have negative consequences which may result in increased risky decision making in later life,[9,10] substance abuse, depression, anxiety and poor school performance,[20]. Suicide or self-harm is the second leading cause of death among 15-29 years,[24] reflecting high suicide rates in South-East Asia,[25,26].

In general, resilience has been defined and described as ability in children and adults to adapt to, adjust to or overcome chronic or acute adversity, providing protection against the development of psychopathology,[27]. It is a multidimensional characteristic that may vary with settings, situations, socio-demographical characteristics, and individually at a more molecular and cellular level and the construct of resilience has been explored in biological, psychosocial, genetic and neurobiological fields,[25]. Sri Lanka which has been through many hardships is a resilient nation; proven through low prevalence of common mental disorders including PTSD during a 3 decade civil war,[28]. However, research on resilience is still scarce in LMIC. Therefore, it is important to identify enabling factors and follow the life-course of resilience of children and adolescents for intervention strategy and policy formulation.

Risk and resilience through a socioecological model in which an adolescent with a particular genome undergoes physical and hormonal transformations within the context of a family, peers, school, work, community and culture, can impact on MNS disorders as an outcome,[15]. Hence, it is crucial to prioritize research on MNS disorders in children and adolescents.

Why Sri Lanka?

Anthropologically, Sri Lanka has a genetically diverse population due to British, Dutch and Portuguese colonisation and Asian immigration. The total population is 20 million with a high literacy rate, and with 33.3% of the population below 18 years of age. It endured a three-decade civil war, and the Asian Tsunami in 2004, resulting in internally displaced people with increased rates of mental disorders,[29]. Eight years post war, it is experiencing demographic transition, rapid globalization, urbanization, and internal/external migration. It has also been proven that children of international labour migrants are at a higher risk of both physical and mental health issues compared to non-migrant families,[30].

The majority of the Sri Lankan population live in the rural sector (77.4%); the urban population share is 18.2%, while 4.4% live in large agricultural estates, considered a separate sector. Urbanization is relatively high in the Western province (38.8%) and very low

For peer review only

in the North Central (4.0%) and North Western (4.1%) provinces. Distribution of facilities and human resources is unequal and specialized services are relatively less in rural areas. Access to health care facilities is more difficult due to greater distances and limited transport services. Poverty is a major problem and education levels are relatively lower in rural areas. Vavuniya has been chosen as a comparative setting as it's a more rural region which was affected by the conflict and war.

There can be an impact of urban/rural differences in the onset of mental health, and therefore we included these two areas to develop the register initially.

Why cohort studies?

Cohort studies in which groups of people are followed up over time are the best method to determining disease incidence and the natural history of a condition. Birth cohort studies can provide important information on risks and resilience across the life course, and they can provide data on childhood, parental and early life environmental characteristics associated with poor health during childhood and in later life. Larger longitudinal birth cohort studies have been conducted within HIC, but these are not generalizable to LMIC,[31]. In order to reduce MNS disorders in adolescents in LMIC, Davidson and colleagues (2015), recommended increased support and development of longitudinal cohort studies. Only a limited number of LMIC birth cohort studies exit, and are located in Guatemala, India, Brazil, Philippines,[15]. Findings from these studies cannot be extrapolated to a country such as Sri Lanka due to the significant socio-cultural differences. Further cohorts throughout South Asia are required to generate data to form new strategies to reduce the incidence and impact of MNS disorders in the region.

Advantages of twin cohort studies

In longitudinal observational studies, confounding poses a considerable threat to the validity of studies aiming to identify causal mechanisms. Twin cohorts can be useful as they allow researchers to understand whether phenotypic relationships are underpinned by shared genetic and or shared environments. Moreover, they can also look at the aetiology of 'environmental variables' and the etiological relationship between these environments and outcomes of interest. A number of studies have found evidence of genetic influences on environmental variables such as stressful life events,[32] a phenomena known as gene-environment correlation,[33]. This additional etiological information can help us to understand more about the causal relationships' phenotypes and environments and phenotypes of interest.

As twinning is largely unrelated to environmental risk factors such as poverty or social class, they allow generalizable assessments of associations and the ability to evaluate the extent of both genetic and environmental confounding; one of the reasons for the increased popularity of twin studies over the past decades,[34].

For peer review only - http://bmjopen.bmj.com/site/about/guidelines.xhtml

AIMS

1. To establish governance, ethical and logistical systems for the creation this register. To strengthen the ethical framework within the initiative by revising the existing guidelines developed by the IRD.
2. To establish a register of infant, child and adolescent twins, and mothers pregnant with twins in the districts of Colombo (Western Province) and Vavuniya (Northern Province), to be approached for future research projects.
3. Gather preliminary information needed to develop future grant applications.
4. Initiate Patient Public Involvement and Engagement (PPIE) work in Sri Lanka through the organisation of local events such as workshops and meetings with stake holders.
5. Explore the feasibility of establishing similar twin registers in Bangladesh India, and Pakistan by initiating small scale exploratory studies.
6. Bring together named investigators to from key long-term partner organisations in LMICs and HICs, and establish the planning and coordinating units, and Steering Committee. Research, ethics and Patient Participant Involvement and Engagement (PPIE) expert groups will be developed to strategize and plan relevant activities.

The overall objective is to extend the health and social care research carried out in Sri Lanka, especially by the Institute for Research and Development, Colombo (IRD). Our current twin research follows a 9,500 twin pair cohort of adults,[35] in the population based Sri Lankan Twin Registry,[36]. We intend on expanding to infant, child and adolescent twin studies with the initial aim of identifying research themes and questions which focus on aetiology of mental illness and the impact of challenges to early brain development on mental health, taking a strategic life course approach. The IRD has a strong core team with a variety of expertise and has assembled a team of long term collaborators; local, regional and from HICs who have expertise in the three main pillars of this initiative which are: (i) high quality research, (ii) ethics, and (iii) PPIE. Existing North-South and South-South collaborations will be used to expand ongoing capacity building activities in CAMH, twin methods, genetic research, and ethics.

METHODOLOGY

There is no specific hypothesis driven research to be conducted during the first year of establishing the infant, child and adolescent twin register. However, the broader hypothesis is that using classical twin methods can compare similarity of identical and non-identical twins to partition the variation of genetic and environmental determinants of disease and traits.

Exact research projects to be undertaken will be decided by methodology experts within the team (Research Experts Group) based on key questions affecting LMIC. Proposals for future research funding applications will be developed during this period of feasibility ascertainment.

The IRD has research instruments for adults, and children and adolescents which have been adapted to both Sinhala and Tamil languages through previous studies, using a more robust method than conventional translation and back translation,[37]. These instruments will be available for future use in projects proposed through this register.

Study Setting; Colombo and Vavuniya districts of Sri Lanka.

The proposed new register will complement and supplement the exiting adult twin register in Colombo district.

Study population

Colombo district is predominantly urban (80%) and has 10% of the Sri Lankan population (2,324,349). The ethnic distribution is 76% Sinhala, 12% Tamil and 11% Moor. 15% of the district population is concentrated in the greater Colombo area where the ethnic distribution is different; 39% Moor, 33% Tamil and 25% Sinhalese. The Vavuniya district has predominantly a rural population (80%) of 172,000, and 83% Tamil, 10% Sinhala and 6% Moor,[38]. These new cohorts provide a good ethnic and urban/rural mix. Vavuniya has been chosen as a comparative setting as it's a more rural region which was affected by the conflict and war.

Inclusion and exclusion

All twins living in Colombo and Vavuniya districts below the age of 18 years will be eligible and have the same opportunity to be registered or not, based on their, or their parents' or guardians' (for minors) wishes. All mothers pregnant with twins will be eligible to register.

Approaches to identification and recruitment

There will be no recruitment for specific research but only registration in the data base. Twins and their parents, and mothers pregnant with twins will be contacted for consent to be included in the register, and to be approached for future research. Data-base registration will not automatically mean involvement or mandatory participation in research. The recruitment phase will last one year only.

All future projects will be first reviewed and approved by the experts in ethics within the project before proceeding to official ethical review by relevant external ethics review committees.

Twins and their parents will be contacted for consent to be included in the register, and to be approached for future research. Data-base registration does not automatically mean involvement in research or involve mandatory participation in research. To recruit twins and to maximise the outcome of this feasibility study, we decided on 4 source populations detailed below.

For peer review only - http://bmjopen.bmj.com/site/about/guidelines.xhtml

Source population A: existing twins from the adult twin registry

Twin off-spring are more common among dizygotic twins compared with singletons and monozygotic pairs,[39]. The adult population-based twin register in Colombo comprises 9500 twin pairs of which 4112 twin pairs are of childbearing age. Of the 4112 approximately 2% would be predicted to have DZ twins (N=82). There are also around 1000 pairs from Colombo up to the age 18 voluntarily registered since establishing the adult register. A dedicated team of research assistants will contact these adult twins by phone and postal methods to determine twin off-springs' aged up to 18 years and invite them to register. Ethics approval and consent has been obtained previously for contacting these twins.

Source population B: maternity hospitals

Almost all deliveries take place in major hospitals in Sri Lanka. We will determine the feasibility of using existing hospital records (in Colombo and Vavuniya) from teaching and maternity hospitals to identify new-born twins and mothers attending antenatal clinics pregnant with twins.

Additionally, we will hold discussions with hospital management on feasible variables for data collection, and using anonymised data from the routine health records, assess its completeness and quality. Potential future data to be collected are detailed in Table 1.

Source Population C: field visits

We shall work in collaboration with the Family Health Bureau which is the central organization of the Sri Lankan Ministry of Health responsible for Maternal and Child Health Services. The front-line health worker providing domiciliary care to mothers and children within the community is the Public Health Midwife (PHM). They work within a well demarcated area having a population ranging from about 2000 to 5000 and conduct regular field visits. We will explore the feasibility of identifying twins by PHM to invite for registration.

Source population D (Schools)

With the support of National Institute for Education and Ministry of Education Sri Lanka, we will conduct a feasibility study of tracing twins through a school survey. There are 584 government schools in both districts and will be possible to identify twins and obtain information about the number of twin pairs in each school, and feasibility of recruitment and engagement. Through the schools we will contact the parents of those twins and provide the information leaflet to register their twin children.

Data to be collected

The primary data collected during the establishment of the register are confined to basic demographic information such as names, dates of birth and contact details. However, information on the possibility of collecting various types of data for future projects will be explored. We will look at the potential data available from school records such as academic performance, entry behaviour assessments administered by Grade 1 upon entry to school.

For peer review only

As well as follow-up on their competencies using criteria developed by National Institute of Education, up-to grade 5, and academic attainment through school reports. Additionally, we will also explore the feasibility of obtaining data such as weight trends over the years, immunization status, and any relevant data collected by the PHM. With the permission from the hospitals and pregnant mothers we shall also assess the possibility of obtaining recorded data (see Table 1 for details).

Table 1. ***Potential data to be collected in future projects***

**Data from hospitals**	**Data up to the date of delivery**<br>• Expected date of delivery and actual date delivered<br>• Medical and obstetrics related data (maternal complications)<br>• Ultrasound scan data<br>• Method of delivery (normal / instrumental / section)<br>• Neonatal complications<br>• Placentation / zygosity<br>• Birth weight / length / head circumference / Apgar score<br><br>**Post delivery**<br>Infant related<br>• Placentation / zygosity<br>• Neonatal complications<br>• Birth weight / length / head circumference / Apgar score<br>• Weight gain<br>• Milestones<br>• Breast feeding<br>• Bonding<br><br>Maternal<br>• Socio demographic data<br>• Type of conception (normal / IVF)<br>• Mood (happiness / sadness)<br>• Maternal nutrition related data<br>• Maternal mental health data<br>• Post-natal complications<br><br>Maternal and Paternal<br>• Willingness to give consent for bio specimens of the infant<br>•

For peer review only

**Data from Primary Health Care workers in the field.**<br><br>**Data will be collected from clinics / house visits.**	**Twin related**<br><u>From health care records available</u><br>• Birth weight / length / head circumference / Apgar score<br>• Weight trends over the years<br>• Immunization history<br>• Health care / illness history including developmental delays<br><br>**Measurements done at home**<br>• Data through questionnaires for:<br>○ Mental health of child<br>○ Cognition of child<br>○ Nutrition related<br><br>**Data from Parents**<br>• Maternal and paternal data as mentioned above<br>• Qualitative interviews from twin's parents:<br>○ including beliefs attitudes and issues about twins<br>○ challenges in rearing twins<br>○ financial implications compared to singletons
**Data from Schools**	**From available school records**<br>• Entry behaviour assessment at Grade 1<br>• Academic attainments indicated through school reports<br>• Records of competency development of children during the primary cycle.<br><br>**Teacher related**<br>• If the twins are in the same school or class<br>• Engagement with peers<br>• Mental health of child

For peer review only

Sample size

Sample size is irrelevant as this is a not a specific research study, however we shall recruit as many twins as possible in both districts. It is also worth noting that twinning rate in Sri Lanka is 10 per 1000 live births,[40]. The Colombo district has 40,000 births per year, with an estimated number of 7,000 twin pairs under the age of 18 years (400 x 18), and the Vavuniya district 4,000 births per year and 700 twin pairs, up to the age of 18 years. Therefore, any future research will have adequate sample sizes depending on the design.

ETHICAL CONSIDERATIONS

Ethics approval

Ethics approval for this study was obtained from the Ethics Review Committee of Sri Lanka Medical Association of Sri Lanka (ref no: ERC/17-025) and the Keele University's Ethical Review Panel (ref no: ERP3157).

To ensure ethical conduct of the highest standards, by both an internal project governance system and an Ethics Experts Group (EEG) consisting of experts in ethics within this project team, and external procedural ethics review which will seek to achieve an appropriate and justifiable balance between respect for participant autonomy, harm vs benefit, risk and social good.

The key ethical sensitivities arising from future research will focus on participants being a vulnerable group (minors, some having mental illness), involving proxy consent, potential for future complex genetic and omics research, sensitive personal data storage, confidentiality, anonymity and use of and access to data, and international collaborations in a different culture.

Managing the involvement of this vulnerable group

Carrying out research among vulnerable populations needs to be handled with extra caution due to the inherent dilemma in such research. We have taken a proactive approach by setting up the EEG, to provide ethical oversight in all activities, to ensure the right blend of scientific rigor and ethics, while also providing a platform to peer review international collaborative research projects. Protection of twins'/parental interests will be ensured for example by avoiding undue incentives to participate, mechanisms to voice concerns, ensuring awareness of the right to withdraw. The EEG will also set up an internal system of governance to regulate the use of the registry that will work in conformity with local ethical review practices.

For all future research projects, specific ethics review and approval will be obtained from relevant ethics review committees (ERC). We will obtain specific ERC approval and consent from participants for any research involving collection and storage of human material. Data collected will be pseudo anonymised but remain linked to participants using a unique identification number.

The IRD has existing guidelines which have been published,[41] and contain the existing policies and guidelines on access to and sharing data/material. It addresses specific concerns when involving human participants and potential use of human tissue and data collection, documentation, storage and access. These guidelines will be revised and updated accordingly.

For peer review only - http://bmjopen.bmj.com/site/about/guidelines.xhtml

In addition, in developing the governance structure we will also incorporate the CIOMS guidelines for future research: more specifically guideline 12; a governance structure in place to ensure the appropriate use of stored data,[42] which is particularly relevant to the proposed work will be considered.

Consent and information provision

Consenting to participate in a cohort study is a long-term commitment, often an uncertain one when specific use of data is not known in advance. Including children and minors in such a project requires parental consent but raises challenges as children grow into mature minors and adults. The EEG will give careful thought to how ongoing consent, assent, and re-consenting will be undertaken as children develop into autonomous persons who can engage in, and make decisions about, research participation. Potential participants will be contacted for consent for register inclusion, but data-base registration does not mandate research participation; appropriate consenting processes will be developed to ensure optimal balance between respect for participants' autonomy and overburdening with individual participation requests. Participants will be informed that registration is entirely voluntary, and that there is no obligation for them to take part. Incentives or payments will not be provided for registering in the twin database.

We shall consult the EEG on how best to contact and obtain assent/consent from twins and their parents. Information leaflets (which have received ethical approval) will be provided to all potential participants. The leaflets will emphasise the database registration but does not involve mandatory participation in research. Future research will need separate ethics clearance from the country of origin and Sri Lanka.

Data Storage, sharing, access confidentiality and anonymity

Existing IRD institutional policies will be followed to ensure secure data storage and usage to guarantee the obligations of confidentiality and anonymity towards participants. IRD will have ownership of the data collected in future research projects. We will only share anonymised data with academics in the future, however they will not have direct access to the IRD twin data bases. Personal data of participants will be stored in a confidential, password protected databases accessible by only the IRD team. Any documents that contain non-anonymized data will be stored in line with UK Data Protection Act (1998). Consent forms will be kept in a separate location to the research data used for analysis so that no linkage can occur. All datasets and databases will be stored on a secure, password protected computer in IRD's secured data room. Backups of this data will be done regularly and stored along with any hard copy information in a locked filling cabinet of the data room.

For security and confidentiality purposes external access to the register will not be allowed. However, we will share anonymous data for higher degree programmes (in Sri Lanka, Kings

For peer review only - http://bmjopen.bmj.com/site/about/guidelines.xhtml

College and Keele University, UK); the data sharing policy based on existing institutional policies will be agreed at the outset.

Public involvement

This is a unique opportunity to benefit LMIC, and HIC through pioneering work in PPIE which is a relatively novel concept in Sri Lanka, and South Asia. Evidence from the UK demonstrates that the quality of research is better, and the likelihood of successful recruitment and implementation of the findings is improved when patients and the public are involved in research,[43].

The IRD in collaboration with the Sri Lankan Medical Association and Keele University, UK held a one-day pre-congress workshop at the 131st Anniversary International Medical Congress of the Sri Lanka Medical Association. Resource persons from IRD, Keele University and other guest lectures from Sri Lanka who are experts in PPIE shared their experience and knowledge with 72 workshop participants. This workshop was pre-planned and funded through MRC, UK grant funding obtained for this study.

We aim to establish a culture of PPIE in Sri Lanka, working with both academic colleagues and lay members; in this instance specifically with twins and their parents. Recruiting lay members to a PPIE group to oversee the work described and work with academics to plan and develop future research questions. Training and culturally adapted materials will enhance their engagement. Engaging more professionals/celebrities (parents of twins or twins themselves) in PPIE activities will increase awareness of twin specific issues from a sociological perspective.

Testing the feasibility of establishing regional twin registers

We shall also initiate a limited amount of feasibility/exploratory studies with our partner institutions Bangladesh, India, and Pakistan. Feasibility proposals submitted by these institutions will be reviewed by the Steering Committee and experts in ethics within this group, and successful applications will be provided limited seed funding through the Institute of Research & Development, Colombo through allocated funds within the MRC grant for this study. Institute for Mind and Brain was successful in obtaining seed funding to carry out an initial small-scale prevalence study in the local area of Wadakkanchery, Kerala, India in order to estimate the incidence of infant, child and adolescent twins in the local school population. This will be the first step in planning to establish a confidential twin registry in the State of Kerala. The team will also conduct a qualitative study to test the feasibility of creating the Kerala twin registry.

For peer review only - http://bmjopen.bmj.com/site/about/guidelines.xhtml

Capacity building

Capacity building is a moral and ethical obligation of externally funded international collaborative research. It will also be bidirectional and collaborative, to advance the local and global agenda is a major component of the project. We have assembled world leading researchers in research methods, ethics, PPIE, and grant development. Ongoing and newly proposed collaborative courses and workshops conducted IRD and this project's collaborators will be open to junior colleagues proposed by the co-investigators. We shall initiate ethics and twin research specific capacity building process in both HIC and LMIC as well, through introductory and advanced courses on twin methods, further strengthening the early and mid-career capacity enhancement by offering the opportunity to work with expert groups. Capacity building benefit specifically on ethics will include enhanced training and knowledge on vulnerable groups (minors, those with mental illness), proxy consent, international collaborations. Funding for such fellowships will be obtained through future grant applications. Capacity building of junior academics across the partnership will develop future research leaders and core skills on child/adolescent mental health and twin research methods.

Dissemination plan

Engagement, communication and dissemination of research and related activities beyond academia are a priority at the IRD. Since its inception, the IRD worked closely with academic partners, policymakers, the public and the electronic/print media. We will promote twin research through electronic media and print, especially using our own trilingual research and science magazine "Gaveshana". Gaveshana is published quarterly by the IRD and approved by the Ministry of Education for distribution to schools. Scientific publications in high impact international journals will allow the sharing of knowledge through a wider network. The initiative and the strategy to work beyond twin methods will be widely publicised to attract a wider research community.

TARGETED OUTCOMES OF THE PROJECT AT THE END OF ONE YEAR

Outputs:

1. A sampling frame (the register) of new-born/child/adolescent twins and mothers pregnant with twins with consent to invite for future research.

2. Identification of a core set of potential data variables which are of sufficient quality for research and are feasible to extract from routine records on children's health and education.

3. Development of key research proposals, taking into consideration research gaps in child and adolescent mental health research.

For peer review only - http://bmjopen.bmj.com/site/about/guidelines.xhtml

4. Revision of the existing IRD ethics and governance guidelines.

5. PPIE related wok which will include a twin gathering event organized to initiate the process of developing networks and engaging twins in twin research.

DISCUSSSION

The proposed new register will complement and supplement the exiting adult twin register in Colombo district.

This unique register in a LMIC will increase the volume of high-quality research and generate a wealth of longitudinal data on mental health and other disciplines. Phenotypic, biological, and anthropometric data, physical measures and social data will allow the studying of health and wellbeing throughout the life course beyond mental health. It will also take cross cultural research into a global context and will provide a better understanding of the mechanisms and dimensions that underlie vulnerability and resilience to mental illness, as well as the personal, social and economic impact of mental illness as a consequence of a wider ranging external drives and risk factors. It will also popularise twin research methods, provide capacity enhancement for junior academics and advances on ethics and PPIE in Sri Lanka and within the region.

The use of the co-twin control design (using differences within twin pairs to examine the association between a putative environmental risk factor and an outcome variable) is extremely efficient for examining risk factor–outcome associations compared to unmatched designs, and through intervention and non-intervention studies offers many novel utilities for cutting edge research: genomics, genetic approaches for prevention,[44] therapy genetics,[45] and studying genetic determinants of response to pharmacological, as well as psychotherapeutic treatment,[46]. Involving twins in RCTs and randomising concordant MZ twins to two arms can control for many of the potential confounding factors, especially genetic makeup due to matching,[47]. It permits large scale epidemiological studies, combining twins with age/gender matched non-twin samples, of both mental disorders and normo-typic behaviour.

This initiative provides capacity building in ethics for LMIC and UK researchers, especially on issues related to cutting edge collaborative research in LMIC. Capacity building benefits specifically on ethics will include enhanced training and knowledge on vulnerable groups (minors, those with mental illness), proxy consent, and international collaborations.

Academics in other disciplines will benefit in this endeavour as well. This will be two-fold; firstly, research beyond mental health, and secondly beyond twin research: the collection of a wealth of longitudinal phenotypic, biological, anthropometric and physical measures as

well as economic, social and psychological data on families with twins. It also enables pioneering education research.

The initiative has implications for foetal medicine because twins are associated with prematurity, low birth weight, greater intra-uterine growth retardation, more birth defects, birth complications and neonatal mortality.

Social value arising from the project is through the potential impact on many domains. Potential societal impact will come from highlighting the additional economic, social, and psychological impact of being a twin, highlighting twins as a unique societal stratum and as a natural laboratory whose research participation has two-way benefits, and therefore create strong awareness among twins, public and policy planners on the above issues.

Since there is a constellation of exposures that put adolescents at risk of MNS disorders and many of which may be simultaneous outcomes of other exposures, research should, measure multiple factors,[15]. Therefore, the proposed work will offer a significant opportunity for future LMIC research in diverse disciplines.

A focus on Sri Lankan twins may limit the generalisability of some findings to other populations. However, heritability is population specific and due to genetic differences between populations all ethnic groups; analyses need to be performed in all ethnic groups to understand a trait at a world-wide level. Being the first twin registry in Sri-Lanka will allow answer questions that could not be studied before – and is playing towards genetic samples more representative of the human diversity. Inhabitant of LMIC are often under-represented in genetic studies and therefore the studies arising from this data base will add significant value to the scarce research in LMIC,[48].

Acknowledgments

We would like to acknowledge the other members of the **SEARCH Group**: Prof Aasim Ahmad (The Aga Khan University, Pakistan), Dr. Achala Jayathilake (Post Graduate Institute of Medicine, Sri Lanka), Prof Ajith Nagahawatte (University of Ruhuna, Sri Lanka), Dr. Anant Bhan (Bioethics and Global Health, India), Dr. Chandima Wickramatilake (University of Ruhuna, Sri Lanka), Dr. Gominda Ponnamperuma (University of Colombo, Sri Lanka), Dr. Lavan Selvarathnam (Ministry of Health, Sri Lanka), Prof Preethi Udagama (University of Colombo, Sri Lanka), Dr. Sorcha Uí Chonnachtaigh, Dr. Steven Blackburn, Dr. Tom Shepherd, and Dr. Toby Helliwell from (Keele University, UK). Dr. Sudath Samaraweera (Ministry of Health, Sri Lanka), Dr. Thamasi Makuloluwa (General Sir John Kotelawala Defence University, Sri Lanka), Dr. Yatan Balhara (All India Institute of Medical Sciences, Delhi), and Ms. Ruwini Cooray (Institute for Research & Development).

For peer review only

We would like to acknowledge Dr. Jayanthi Gunesekara (National Institute of Education), Mrs. Jayani Tillakaratne (Ministry of Education), Mr. Saman Indrajith (The Island Newspaper, Sri Lanka). We also highly appreciate the ongoing support from Dr. Nethanjalee Mapitigama, Dr. Kaushalya Kasthuriarachchi, and Dr. Hiranya Jayawickrama of the Family Health Bureau, and Dr. Ruwan Wijayamuni, Chief Medical Officer, Colombo Municipal Council. We would also like to thank higher officials of the Ministry of Health and the Ministry of Education.

We gratefully acknowledge members of the Institute for Research & Development, Dr. Irena Zwierska and Ms. Rebecca Parker at Keele University who have contributed immensely to the preparation and execution of the project.

Funding

This study is supported by a one-year pump priming grant awarded by the Medical Research Council, UK (Grant number: MC_PC_MR/R018448/1). AS currently receives salary support in kind from the Midland Partnership Foundation NHS Trust, UK.

Authors' contributions

All authors contributed to the drafting of original funding application which is the basis of this protocol paper. Athula Sumathipala was responsible for the concept of the application and the initial draft of the paper. Fruhling Rijsdijk, Richard Saffery, Emily Simonoff, Jeffrey M Craig, and Helena M S Zavos were mainly responsible for twin design component. Buddhika Fernando, Jonathan Ives and Athula Sumathipala were mainly responsible for ethics and governance component. Nihal Abeysinghe, Nick Glozier, Asiri P Hewamalage, Kelvin P Jordan, Christian Mallen, Omar Rahman, Shamsa Zafar, Rita Yusuf, Kaushalya Jayaweera, and Lasith Dissanayake were mainly responsible for epidemiology and statistics components. Sunil B De Alwis, Krysia Dziedzic, and Godwin Kodituwakku were mainly responsible for PPIE component while Alka Saxena for genetic component. Lasith Dissanayake and Alina Andras also contributed to obtaining ethical clearance. Kaushalya Jayaweera, Lasith Dissanayake and Athula Sumathipala contributed at all stages of planning, writing & editing the article based on the original funding application. As this is a protocol paper there was no acquisition of data or analysis and interpretation of data. All authors contributed to different drafts of the paper and finally approved the manuscript for submission.

Competing interests

The authors declare that they have no competing interests.

For peer review only

For peer review only - http://bmjopen.bmj.com/site/about/guidelines.xhtml

References:

1 Georgieff MK. Nutrition and the developing brain: Nutrient priorities and measurement. In: *American Journal of Clinical Nutrition*. 2007. 614–20. doi:85/2/614S [pii]

2 Kessler RC, McLaughlin KA, Green JG, *et al.* Childhood adversities and adult psychopathology in the WHO world mental health surveys. *Br J Psychiatry* 2010;**197**:378–85. doi:10.1192/bjp.bp.110.080499

3 Read J, Bentall RP, Fosse R. Time to abandon the bio-bio-bio model of psychosis: Exploring the epigenetic and psychological mechanisms by which adverse life events lead to psychotic symptoms. In: *Epidemiology and Psychiatric Sciences*. Cambridge University Press 2009. 299–310. doi:10.1017/S1121189X00000257

4 Green B, Friedman M, de Jong J, *et al. Trauma Interventions in War and Peace: Prevention, Practice, and Policy*. 2003. https://books.google.com/books?hl=en&lr=&id=10yifSbPQuYC&oi=fnd&pg=PA1&ots=WUbnI68X_D&sig=rBNjCKOSDjL8dvVD0J7LtQpjO7o (accessed 3 Aug 2017).

5 Betancourt TS, Khan KT. The mental health of children affected by armed conflict: protective processes and pathways to resilience. *Int Rev Psychiatry* 2008;**20**:317–28. doi:10.1080/09540260802090363

6 Giaconia RM, Reinherz HZ, Hauf AA, *et al.* Comorbidity of Substance Use and Post-Traumatic Stress Disorders in a Community Sample of Adolescents. *Am J Orthopsychiatry* 2000;**70**:253–62. doi:10.1037/ h0087634

7 Patton GC, Coffey C, Romaniuk H, *et al.* The prognosis of common mental disorders in adolescents: a 14-year prospective cohort study. *Lancet (London, England)* 2014;**383**:1404–11. doi:10.1016/S0140-6736(13)62116-9

8 Neufeld SAS, Dunn VJ, Jones PB, *et al.* Reduction in adolescent depression after contact with mental health services: a longitudinal cohort study in the UK. *The lancet Psychiatry* 2017;**4**:120–7. doi:10.1016/S2215-0366(17)30002-0

9 Hallfors DD, Waller MW, Ford CA, *et al.* Adolescent depression and suicide risk: Association with sex and drug behavior. *Am J Prev Med* 2004;**27**:224–31. doi:10.1016/j.amepre.2004.06.001

10 Shrier LA, Harris SK, Sternberg M, *et al.* Associations of depression, self-esteem, and substance use with sexual risk among adolescents. *Prev Med (Baltim)* 2001;**33**:179–89. doi:10.1006/pmed.2001.0869

11 Hussong AM. Perceived Peer Context and Adolescent Adjustment. *J Res Adolesc* 2000;**10**:391–415. doi:10.1207/SJRA1004_02

12 Newman BM, Newman PR, Griffen S, *et al.* The relationship of social support to depressive symptoms during the transition to high school. *Adolescence* 2007;**42**:441–

For peer review only - http://bmjopen.bmj.com/site/about/guidelines.xhtml

59.http://search.proquest.com/openview/a934da86c87c826c5935e63dd27cf611/1?pq-origsite=gscholar&cbl=41539 (accessed 3 Aug 2017).

13 Peirce R, Frone M, Russell M, *et al.* A Longitudinal Model of Social Contact, Social Support, Depression, and Alcohol Use. *Heal Psychol* 2000;**19**:28–38. doi:10.1037/0278-6133.19.1.28

14 Forsyth JK, Ellman LM, Tanskanen A, *et al.* Genetic risk for schizophrenia, obstetric complications, and adolescent school outcome: Evidence for gene-environment interaction. *Schizophr Bull* 2013;**39**:1067–76. doi:10.1093/schbul/sbs098

15 Davidson LL, Grigorenko EL, Boivin MJ, *et al.* A focus on adolescence to reduce neurological, mental health and substance-use disability. *Nature* 2015;**527**:S161–6. doi:10.1038/nature16030

16 Patel V, Flisher AJ, Hetrick S, *et al.* Mental health of young people: a global public-health challenge. *Lancet* 2007;**369**:1302–13. doi:10.1016/S0140-6736(07)60368-7

17 Kieling C, Baker-Henningham H, Belfer MM, *et al.* Child and adolescent mental health worldwide: evidence for action. *Lancet (London, England)* 2011;**378**:1515–25. doi:10.1016/S0140-6736(11)60827-1

18 Sharan P, Gallo C, Gureje O, *et al.* Mental health research priorities in low- and middle-income countries of Africa, Asia, Latin America and the Caribbean. *Br J Psychiatry* 2009;**195**:354–63. doi:10.1192/bjp.bp.108.050187

19 WPA, WHO, IACAPAP. Atlas: Child and Adolescent Mental Health Resources: Global Concerns, Implications for the Future. 2005. doi:10.3109/08039487509097356

20 Ranasinghe S, Ramesh S, Jacobsen KH. Hygiene and mental health among middle school students in India and 11 other countries. *J Infect Public Health* 2016;**9**:429–35. doi:10.1016/j.jiph.2015.11.007

21 WHO. Mental Health Action Plan 2013-2020. *WHO Libr Cat DataLibrary Cat Data* 2013;:1–44. doi:ISBN 978 92 4 150602 1

22 Deykin EY, Buka SL. Prevalence and risk factors for posttraumatic stress disorder among chemically dependent adolescents. *Am J Psychiatry* 1997;**154**:752–7. doi:10.1176/ajp.154.6.752

23 Perkonigg A, Kessler RC, Storz S, *et al.* Traumatic events and post-traumatic stress disorder in the community: prevalence, risk factors and comorbidity. *Acta Psychiatr Scand* 2000;**101**:46–59. doi:10.1034/j.1600-0447.2000.101001046.x

24 WHO. WORLD HEALTH STATISTICS - MONITORING HEALTH FOR THE SDGs. *World Heal Organ* 2016;:1.121. doi:10.1017/CBO9781107415324.004

25 Rutten BPF, Hammels C, Geschwind N, *et al.* Resilience in mental health: Linking psychological and neurobiological perspectives. Acta Psychiatr. Scand. 2013;**128**:3–20. doi:10.1111/acps.12095

26 WHO. *Mental Health Status of Adolescents in South-East Asia : Evidence for Action.*

For peer review only

For peer review only - http://bmjopen.bmj.com/site/about/guidelines.xhtml

World Health Organization, South-East Asia Regional Office 2017. http://apps.who.int/iris/bitstream/10665/254982/1/9789290225737-eng.pdf?ua=1&ua=1&ua=1&ua=1

27 Bonanno GA. Loss, Trauma, and Human Resilience: Have We Underestimated the Human Capacity to Thrive after Extremely Aversive Events? Am. Psychol. 2004;**59**:20–8. doi:10.1037/0003-066X.59.1.20

28 Institute for Research and Development. *National Mental Health Survey Report*. Colombo: 2007.

29 Siriwardhana C. Mental health of displaced and returnee populations: Insight from the Sri Lankan post-conflict experience. *Confl Health* 2015;**9**:22. doi:10.1186/s13031-015-0049-2

30 Wickramage K, Siriwardhana C, Vidanapathirana P, *et al.* Risk of mental health and nutritional problems for left-behind children of international labor migrants. *BMC Psychiatry* 2015;**15**:39. doi:10.1186/s12888-015-0412-2

31 Baxter AJ, Patton G, Scott KM, *et al.* Global Epidemiology of Mental Disorders: What Are We Missing? *PLoS One* 2013;**8**:e65514. doi:10.1371/journal.pone.0065514

32 Kendler KS, Baker JH. Genetic influences on measures of the environment: A systematic review. Psychol. Med. 2007;**37**:615–26. doi:10.1017/S0033291706009524

33 Scarr S, McCartney K. How People Make Their Own Environments: A Theory of Genotype - Environment Effects. *Child Dev* 1983;**54**:424. doi:10.2307/1129703

34 Gjerde LC, Czajkowski N, Roysamb E, *et al.* A longitudinal, population-based twin study of avoidant and obsessive-compulsive personality disorder traits from early to middle adulthood. *Psychol Med* 2015;**45**:3539–48. doi:10.1017/S0033291715001440

35 Jayaweera K, Aschan L, Pannala G, *et al.* The Colombo Twin and Singleton Follow-up Study: A population based twin study of psychiatric disorders and metabolic syndrome in Sri Lanka. *BMC Public Health* 2018;**18**:145. doi:10.1186/s12889-017-4992-2

36 Siribaddana SH, Siriwardane WD, Hewage SN, *et al.* Update from Sri Lankan Twin Registry: Establishment of a population-based twin register and ongoing project on common mental disorders, alcohol abuse and suicidal ideations. *Twin Res Hum Genet* 2006;**9**:868–74. doi:10.1375/183242706779462679

37 Sumathipala A, Murray J. New approach to translating instruments for cross-cultural research: A combined qualitative and quantitative approach for translation and consensus generation. *Int J Methods Psychiatr Res* 2000;**9**:87–95. doi:10.1002/mpr.83

38 Department of Census & Statistics. Census of Population and Housing 2012. Department of Census & Statistics and Ministry of Policy Planning and Economic Affairs. 2015. http://www.statistics.gov.lk/PopHouSat/CPH2011/Pages/Activities/Reports/FinalReport/FinalReportE.pdf (accessed 24 Oct 2018).

For peer review only

39 Hoekstra C, Zhao ZZ, Lambalk CB, *et al.* Dizygotic twinning. *Hum Reprod Update* 2008;**14**:37–47. doi:10.1093/humupd/dmm036

40 Medical Statistics Unit. Annual Health Statistics 2015. 2017.

41 Sumathipala A, Siribaddana S. *Research Ethics from a Developing World Perspective*. Vijitha Yapa publishers, Colombo 2003. https://search.informit.com.au/fullText;dn=978230397803004;res=E-LIBRARY (accessed 14 Aug 2017).

42 CIOMS. International Ethical Guidelines for Health-related Research Involving Humans Fourth Edition. Geneva. *Biomed Res* 2016;:1–122.http://www.sciencedirect.com/science/article/B6VC6-45F5X02-9C/2/e44bc37a6e392634b1cf436105978f01

43 Mockford C, Staniszewska S, Griffiths F, *et al.* The impact of patient and public involvement on UK NHS health care: A systematic review. Int. J. Qual. Heal. Care. 2012;**24**:28–38. doi:10.1093/intqhc/mzr066

44 WHO. Community genetic services: report of a WHO consultation on community genetics in low- and middle-income countries. 2010. doi:10.1159/000080789

45 Plomin R, Haworth CM a. Genetics and Intervention research. *Perspect Psychol Sci* 2010;**5**:557–63. doi:10.1177/1745691610383513

46 Eley TC, Hudson JL, Creswell C, *et al.* Therapygenetics: The 5HTTLPR and response to psychological therapy. *Mol Psychiatry* 2012;**17**:236–7. doi:10.1038/mp.2011.132

47 Sumathipala A, Yelland L, Green D, *et al.* Twins as participants in randomised controlled trials: a review of published literature. *Twin Res Hum Genet* 2018.

48 Polderman TJC, Benyamin B, De Leeuw CA, *et al.* Meta-analysis of the heritability of human traits based on fifty years of twin studies. *Nat Genet* 2015;**47**:702–9. doi:10.1038/ng.3285

For peer review only

BMJ Open

Protocol for establishing a child and adolescent twin register for mental health research, and capacity building in Sri Lanka and other low and middle-income countries in South Asia

Journal:	*BMJ Open*
Manuscript ID	bmjopen-2019-029332.R2
Article Type:	Protocol
Date Submitted by the Author:	07-Aug-2019

Complete List of Authors:	Jayaweera, Kaushalya; Institute for Research & Development, Section of Epidemiology<br>Craig, Jeffrey; Deakin University, School of Medicine<br>Zavos, Helena; King's College London, MRC Social, Genetic, and Developmental Psychiatry Centre<br>Abeysinghe, Nihal; Institute for Research & Development in Health and Social Care, Sri Lanka, ;<br>De Alwis, Sunil; Ministry of Health, Nutrition and Indigenous Medicine, Medical Services<br>Andras, Alina; Keele University, ;<br>Dissanayake, Lasith; Institute for Research and Development, Epidemiology<br>Dziedzic, Krysia; Arthritis Research Campaign National Primary Care Centre<br>Fernando, Buddhika; Research Institute for Primary Care and Health Sciences, ;<br>Glozier, Nick; University of Sydney Brain and Mind Research Institute,<br>Hewamalage, Asiri; Ministry of Health and indegenous Medicine, Sri Lanka, Department of Health;<br>Ives, Jonathan; University of Bristol, Centre for Ethics in Medicine<br>Jordan, Kelvin; Keele University, Primary Care & Health Sciences<br>Kodituwakku, Godwin; Institute for Research & Development in Health and Social Care, Sri Lanka,<br>Mallen, Christian; Keele University, Arthritis Research UK Primary Care Centre<br>Rahman , Omar; Independent University, Public Health<br>Zafar, Shamsa; Air University,<br>Saxena, Alka; Guy's and Saint Thomas' Hospitals NHS Trust, Biomedical Research Centre;<br>Rijsdijk, Fruhling; King's College London, Social, Genetic, and Developmental Psychiatry Centre<br>Saffery, Richard; Murdoch Childrens Research Institute, Cancer and Disease Epigenetics; University of Melbourne, Paediatrics<br>Simonoff, Emily; King's College London, Institute of Psychiatry, Child & Adolescent Psychiatry<br>Yusuf, Rita; Independent University, School of Life Sciences;<br>Sumathipala, Athula; Institute for Research & Development, Research Institute for Primary Care & Health Sciences ; Keele University

	Staffordshire,
**Primary Subject Heading**:	Epidemiology
Secondary Subject Heading:	Genetics and genomics, Patient-centred medicine, Global health
Keywords:	Sri Lanka, Twins, Infant child and adolescents, Mental health, Cohort, South Asia, Low and Middle Income Countries (LMIC)

SCHOLARONE™
Manuscripts

Protocol for establishing a child and adolescent twin register for mental health research, and capacity building in Sri Lanka and other low and middle-income countries in South Asia

Kaushalya Jayaweera[1] & Jeffrey Craig[2], Helena Zavos[3], Nihal Abeysinghe[1], Sunil De Alwis[4], Alina Andras[5], Lasith Dissanayake[1], Krysia Dziedzic[5], Buddhika Fernando[1,5], Nick Glozier[6], Asiri Hewamalage[4], Jonathan Ives[7], Kelvin Jordan[5], Godwin Kodituwakku[1], Christian Mallen[5], Omar Rahman[8], Shamsa Zafar[9], Alka Saxena[10], Fruhling Rijsdijk[11], Richard Saffery[12], Emily Simonoff[13], Rita Yusuf[8], Athula Sumathipala[1,5*]. On behalf of SEARCH group

Corresponding Author* - Prof. Athula Sumathipala, Keele University, Staffordshire, ST5 5BG, UK. a.sumathipala@keele.ac.uk, +441782732000

1 Institute for Research and Development in Health and Social Care, Colombo, Sri Lanka

2 Faculty of Health, Deakin University, Australia

3 Department of Psychology, Institute of Psychiatry, Psychology & Neuroscience, Kings College London, UK

4 Ministry of Health and Indigenous Medicine, Colombo, Sri Lanka

5 Research Institute for Primary Care & Health Sciences, Faculty of Medicine & Health Sciences, Keele University, UK.

6 Brain and Mind Centre, University of Sydney, Australia

7 Center for Ethics and Medicine, University of Bristol, UK

8 Independent University, Bangladesh

9 Health Services Academy, Pakistan

10 NIHR Biomedical Research Centre, Guy's Hospital, Kings College London, UK

11 Social Genetic and Developmental Research Centre, Institute of Psychiatry, Psychology & Neuroscience, King's College London, UK

12 Murdoch Children Research Institute, Victoria, Australia

13 Department of Child and Adolescent Psychiatry, King's College London, Institute of Psychiatry, Psychology & Neuroscience, and NIHR Biomedical Research Centre for Mental Health, UK

For peer review only

ABSTRACT

Introduction

Worldwide, 10-20% of children and adolescents experience mental health conditions. However, most such disorders remain undiagnosed until adolescence or adulthood. Little is known about the factors that influence mental health in children and adolescents, especially in low and middle-income countries (LMIC), where environmental threats, such as poverty and war, may affect optimal neurodevelopment. Cohort studies provide important information on risks and resilience across the life course by enabling tracking of the effects of early life environment on health during childhood and beyond. Large birth cohort studies, including twin cohorts which can be aetiologically informative,

have been conducted within high income countries, but are not generalizable to LMIC. There are limited longitudinal birth cohort studies in LMIC.

Methods

We sought to enhance the volume of impactful research in Sri Lanka by establishing a Centre of Excellence for cohort studies. The aim is to establish a register of infant, child and adolescent twins, including mothers pregnant with twins, starting in the districts of Colombo (Western Province) and Vavuniya (Northern Province). We will gain consent from twins or parents for future research projects. This register will provide the platform to investigate the aetiology of mental illness and the impact of challenges to early brain development on future mental health. Using this register we will be able to conduct research which will (1) expand existing research capacity on child and adolescent mental health and twin methods; (2) further consolidate existing partnerships; (3) and establish new collaborations. The initiative is underpinned by three pillars: high quality research, ethics, and patient and public involvement and engagement.

Ethics and dissemination

Ethics approval for this study was obtained from the Ethics Review Committee of Sri Lanka Medical Association and Keele University's Ethical Review Panel. In addition to journal publications a range of PPIE activities have been conducted.

Article summary: Strengths and Limitations

- The only population-based register of child and adolescent twins established in a Low and Middle Income Country
- The project includes data base development, ethics development and Public and Patients Involvement and Engagement
- The cohort compliments the existing adult twin register in Colombo Sri Lanka and 15 years of twin research experience
- The focus on Sri Lankan twins may limit the generalisability of some findings to other populations

Keywords

Sri Lanka, twins, infant, children, adolescents, mental health, early life origins, cohort

BACKGROUND

A substantial proportion of the world's health problems in both high-income countries (HICs) and low and middle-income countries (LMICs) arises from mental, neurological, and substance use conditions,[1]. Worldwide, 10-20% of children and adolescents experience mental health conditions, and many transition into adulthood undiagnosed and continue to experience these disorders,[2]. Child and adolescent mental health is especially important in LMIC, as treatment and prevention resources are generally low in comparison to adults,[3]. A number of prenatal, perinatal and postnatal factors have been associated with increased risk of neurodevelopmental disorders and mental health conditions across the life course,[4–6]. However, much of this research has been based in HIC. By establishing a child and adolescent twin cohort, we will establish a resource which will enable studies to conduct research on the impact of early life risk factors on the development of neurodevelopment and mental health conditions in a LMIC.

Challenges to early brain development take place in intrauterine life and continue throughout childhood and adolescence. Research has shown a number of key risk factors in the foetal and early postnatal life as being associated with a range of neurodevelopment and mental health conditions,[4]. Research in HICs have suggested that nutrients and growth factors regulate brain development during foetal and early postnatal life and rapidly developing brain is more vulnerable to nutrient insufficiency,[7]. Other factors such as low birth weight and hypoxia, which are more common in LMICs,[8], are among the environmental factors that have been consistently associated with a range of neurodevelopmental and mental health conditions across the lifespan.

For peer review only - http://bmjopen.bmj.com/site/about/guidelines.xhtml

Disasters, both natural and war, are well known causes of a variety of psychological and psychiatric conditions [9]. Research has suggested that children and adolescents exposed to war related traumatic events are affected mental health distress, and in certain cases long term psychopathology,[10]. There is also a strong relationship between adolescents exposed to trauma and suffering from PTSD and substance abuse. Studies have shown that more than half of the young people with PTSD subsequently develop substance abuse problems,[11]. These publications have identified the need for more research in LMICs.

WHO, in a survey of mental health research priorities in LMICs including Asia, identified children and adolescents exposed to violence/trauma as prioritized population groups,[12], whilst risk and resilience can impact on MNS disorders and their outcomes. In both cases there is a significant gap in research in LMICs [13]. Interestingly, there are relatively low rates of common mental disorders among adults in Sri Lanka,[14]. However, limited similar research has been conducted to date on children and adolescents in Sri Lanka.

Cohort studies, in which groups of people are followed up over time, are the best method to determine disease incidence and longitudinal pattern of physical and mental health conditions and the natural history. Cohort studies can provide important information on risks and resilience factors across the life course. They can also provide data on childhood, parental and early life environmental factors associated with poor health during childhood and in later life, and risks and resilience factors across the life course. Large longitudinal birth cohort studies have been conducted within HIC, but are not generalizable to LMIC,[15]. In order to reduce MNS disorders in adolescents in LMIC, Davidson and colleagues,[13] recommend increased support and development of longitudinal cohort studies. Only a limited number of LMIC birth cohort studies exit, and are located in Guatemala, India, Brazil, Philippines,[13]. Findings from these studies are difficult to apply to a country such as Sri Lanka due to significant socio-cultural differences, further cohorts throughout South Asia are required to generate data to form new strategies to reduce the incidence and impact of MNS disorders in the region.

The use of the co-twin control design (using differences within twin pairs to examine the association between a putative environmental risk factor and an outcome variable) is extremely efficient for examining risk factor–outcome associations compared to unmatched designs. And through intervention and non-intervention studies offers many novel utilities for cutting edge research: genomics, genetic approaches for prevention,[16], therapy genetics,[17], and studying genetic determinants of response to pharmacological, as well as psychotherapeutic treatment,[18]. Involving twins in RCTs and randomising concordant MZ twins to two arms can control for many of the potential confounding factors, especially genetic makeup due to matching,[19]. It permits large scale epidemiological studies, combining twins with age/gender matched non-twin samples, of both mental disorders and normotypic behaviour.

In longitudinal observational studies, confounding poses a considerable threat to the validity of studies aiming to identify causal mechanisms. Twin cohorts can be useful as they allow researchers to understand the role of genes and environment on outcomes of interest. In addition, a number of studies have found evidence of genetic influences on environmental variables such as stressful life events,[20], a phenomena know as gene-environment correlation,[21]. This additional etiological information can help us to understand more about the causal relationships, phenotypes and environments, and phenotypes of interest. As twinning is largely unrelated to environmental risk factors such as poverty or social class, twin studies allow generalizable assessments of associations and the ability to evaluate the extent of both genetic and environmental confounding,[22].

Focus on Sri Lanka

Anthropologically, Sri Lanka has a genetically diverse population of 20 million with a high literacy rate, and with 33.3% of the population below 18 years of age. It has endured a three-decade civil war, and the Asian Tsunami in 2004. Ten years post war, it is experiencing demographic transition, rapid globalization, urbanization, and internal/external migration [23].

For peer review only - http://bmjopen.bmj.com/site/about/guidelines.xhtml

The majority of the Sri Lankan population lives in the rural sector (77.4%); the urban population share is 18.2%, while 4.4% live in large agricultural estates, considered a separate sector,[24,25]. Poverty is a major problem and education levels are relatively lower in rural areas,[25]. Vavuniya (Northern Province) has been chosen as a comparative setting to Colombo (Western Province) as it is a more rural region directly affected by civil war. Urban/rural differences can have an impact of on the onset of mental health,[25], and therefore we included these two areas to develop the register initially.

Aims

The overall objective is to extend the health and social care research carried out in Sri Lanka, especially by the Institute for Research and Development in Health and Social Care, Colombo (IRD). Our current twin research follows a 9,500 twin pair cohort of adults,[14] in the population-based Sri Lankan Twin Registry,[26]. The IRD has a strong core team with a variety of expertise and has assembled a team of long-term collaborators; local, regional and from HICs who have expertise in the three main pillars of this initiative which are: high quality research, ethics, and PPIE. We aim (1) to establish a register of infant, child and adolescent twins, and mothers pregnant with twins in the districts of Colombo and Vavuniya, to be approached for future research projects. (2) Gather preliminary information needed to develop future grant applications. (3) Initiate and conduct Patient and Public Involvement and Engagement (PPIE) work in Sri Lanka through the organisation of local events such as workshops and meetings with stakeholders. (4) Bring together investigators from key long-term partner organisations in LMICs and HICs, and establish the planning and coordinating units, and Steering Committee. Research, ethics and Patient Participant Involvement and Engagement (PPIE) expert groups will be developed to strategize and plan relevant activities.

METHODS

The broad hypothesis of this project is to apply classical twin methods and the MZ differences design to understand the prevalence and aetiology of a range of neurodevelopmental and mental health conditions affecting children and adolescents in two districts of Sri Lanka. Specific research projects to be undertaken will be decided by the IRD in consultation with relevant stakeholders in Sri Lanka, and supported by the methodology experts group of SEARCH based on available funding calls.

Study Setting: Colombo and Vavuniya districts of Sri Lanka.

Colombo district is predominantly urban and comprises 10% of the Sri Lankan population. The ethnic distribution is 76% Sinhala, 12% Tamil and 11% Moor. 15% of the district population is concentrated in the greater Colombo area where the ethnic distribution is almost equal in proportion to each other. The Vavuniya district is mainly classified as rural, and the ethnic majority are Tamil (83%)[27]. These new cohorts provide a good ethnic and urban/rural mix. The proposed new register will complement and supplement the exiting adult twin register in Colombo district.

Inclusion and exclusion

All twins below the age of 18 years and mothers pregnant with twins living in Colombo and Vavuniya districts will be eligible, and will have the same opportunity to be registered or not to be registered, based on their, or their parents' or guardians' (for minors) wishes.

Approaches to identification and recruitment

There will be no recruitment for specific research but only registration onto the database. Twins, and their parents, and mothers pregnant with twins will be contacted for consent to be included in the register. Database registration will not automatically mean involvement or mandatory participation in research.

All future projects will be first reviewed by the Ethics Experts Group in SEARCH project before proceeding to external ethical review in Sri Lanka and the country of external collaboration. To recruit twins and to maximise the outcome of this feasibility study, we decided on four source populations detailed below:

Existing twins from adult twin registry

Twin offspring are more common among dizygotic twins compared with singletons and monozygotic pairs,[28]. The adult population-based twin register in Colombo comprises 9500 twin pairs of which

For peer review only - http://bmjopen.bmj.com/site/about/guidelines.xhtml

4112 twin pairs are of child bearing age. Of the 4112 approximately 2% would be predicted to have DZ twins (N=82).There are also around 1000 pairs from Colombo up to the age 18 voluntarily registered since establishing the adult register. We will contact these adult twins by phone and postal methods to determine twin offspring aged up to 18 years and invite them to register. Ethics approval and consent has been obtained previously for contacting these twins,[29].

Maternity hospitals

Almost all deliveries (singletons and higher order multiples) take place in major hospitals in Sri Lanka. We will determine the feasibility of using existing hospital records (in Colombo and Vavuniya) from teaching and maternity hospitals to identify new-born twins and mothers attending antenatal clinics pregnant with twins. Additionally, we will hold discussions with hospital management on feasible variables for data collection, and using anonymised data from the routine health records. Potential future data to be collected are detailed in Table 1.

Field visits

We will work in collaboration with the Family Health Bureau which is the central organization of the Sri Lankan Ministry of Health responsible for Maternal and Child Health Services. The front-line health worker providing domiciliary care to mothers and children within the community is the Public Health Midwife (PHM). They work within a well demarcated area having a population ranging from about 2000 to 5000 and conduct regular field visits. We will explore the feasibility of identifying twins by PHM to invite for registration.

Source population D (Schools)

With the support of the National Institute for Education and Ministry of Education Sri Lanka, we will conduct a feasibility study of tracing twins through a school survey. There are 584 government schools in both districts and will be possible to identify twins and obtain information about the number of twin pairs in each school, and feasibility of recruitment and engagement. Through the schools we will contact the parents of those twins and provide the information leaflet to register their twin children.

Data to be collected

The primary data collected during the establishment of the register are confined to basic demographic information such as names, dates of birth and contact details. Table 1 describes the sources and potential types of data to be collected in future studies. We will look at the potential data available from school records such as academic performance, entry behaviour assessments administered by Grade 1 upon entry to school. As well as follow-up on their competencies using criteria developed by National Institute of Education, up-to grade 5, and academic attainment through school reports. Additionally, we will also explore the feasibility of obtaining data such as weight trends over the years, immunization status, and any relevant data collected by the PHM. With the permission from the hospitals and pregnant mothers we shall also assess the possibility of obtaining recorded data.

*Table 1. **Potential data to be collected in future projects***

**Data from hospitals**	**Data up to the date of delivery**<br>• Expected date of delivery and actual date delivered<br>• Medical and obstetrics related data (maternal complications)<br>• Ultra sound scan data<br>• Method of delivery (normal /instrumental/section)<br><br>**Post delivery**<br>Infant related<br>• Placentation/ zygosity<br>• Neonatal complications<br>• Birth weight/ length/head circumference / Apgar score<br>• Weight gain<br>• Mile stones<br>• Breast feeding<br>• Bonding

For peer review only

	Maternal<br>• Socio demographic data<br>• Type of conception (normal/IVF)<br>• Mood (happiness/sadness)<br>• Maternal nutrition related data<br>• Maternal mental health data<br>• Post-natal complications<br><br>Maternal and Paternal<br>• Willingness to give consent for bio specimens of the infant
**Data from Primary Health Care workers in the field.**<br><br>**Data will be collected from clinics/house visits.**	**Twin related**<br>From health care records available<br>• Birth weight/ length/head circumference / Apgar score<br>• Weight trends over the years<br>• Immunization history<br>• Health care/illness history including developmental delays<br><br>**Measurements done at home**<br>• Data through questionnaires for:<br>○ Mental health of child<br>○ Cognition of child<br>○ Nutrition related<br><br>**Data from Parents**<br>• Maternal and paternal data as mentioned above<br>• Qualitative interviews from twin's parents:<br>○ including beliefs attitudes and issues about twins<br>○ challenges in rearing twins<br>○ financial implications compared to singletons
**Data from Schools**	**From available school records**<br>• Entry behaviour assessment at Grade 1<br>• Academic attainments indicated through school reports<br>• Records of competency development of children during the primary cycle.<br><br>**Teacher related**<br>• If the twins are in the same school or class<br>• Engagement with peers<br>• Mental health of child

For peer review only

Sample size

We will recruit as many twins as possible in both districts. The Colombo district has 40,000 births per year, with an estimated number of 7,000 twin pairs under the age of 18 years, and the Vavuniya district 4,000 births per year and 700 twin pairs, up to the age of 18 years.

Testing the feasibility of establishing regional twin registers

We will also initiate feasibility/exploratory studies with our partner institutions Bangladesh, India, and Pakistan. Feasibility proposals submitted by these institutions will be reviewed by the Steering Committee and experts in ethics within this group, and successful applications will be provided limited seed funding through the IRD, Colombo through allocated funds within the MRC grant for this study. Institute for Mind and Brain was successful in obtaining seed funding to carry out an initial small-scale

prevalence study in the local area of Wadakkancherry, Kerala, India in order to estimate the incidence of infant, child and adolescent twins in the local school population. This was the first step in planning to establish a confidential twin registry in the State of Kerala. The team will also conduct a qualitative study to test the feasibility of creating the Kerala twin registry.

Capacity building

Capacity building is a moral and ethical obligation of externally funded international collaborative research. It will also be bidirectional and collaborative, to advance the local and global agenda is a major component of the project. Ongoing and newly proposed collaborative courses and workshops conducted IRD and this project's collaborators will be open to junior colleagues proposed by the co-investigators. We will initiate ethics and twin research specific capacity building process in both HIC and LMIC as well, through introductory and advanced courses on twin methods, further strengthening the early and mid-career capacity enhancement by offering the opportunity to work with expert groups. Funding for such fellowships will be obtained through future grant applications. Capacity building of junior academics across the partnership will develop future research leaders and core skills on child/adolescent mental health and twin research methods.

ETHICS AND DISSEMINATION

Ethical approval for development of a child and adolescent twin registry was obtained from the Ethics Review Committee of Sri Lanka Medical Association (ref no: ERC/17-025) and Keele University's Ethical Review Panel (ref no: ERP3157).

An internal project governance system and an Ethics Experts Group (EEG) consisting of experts in ethics within this project team, and external procedural ethics review will ensure ethical conduct of the highest standards. The key ethical sensitivities arising from future research will focus on participants being a vulnerable group (minors, some having mental illness), involving proxy consent, potential for future complex genetic and omics research, sensitive personal data storage, confidentiality, anonymity and use of and access to data, and international collaborations in a different culture.

Managing the involvement of this vulnerable group

Carrying out research among vulnerable populations needs to be handled with extra caution due to the inherent dilemma in such research. We have taken a proactive approach by setting up the EEG, to provide ethical oversight in all activities, to ensure the right blend of scientific rigor and ethics, while also providing a platform to peer review international collaborative research projects. Protection of twins'/parental interests will be ensured for example by avoiding undue incentives to participate, mechanisms to voice concerns, ensuring awareness of the right to withdraw. The EEG will also set up an internal system of governance to regulate the use of the registry that will work in conform with local ethical review practices.

For all future research project specific ethics review and approval will be obtained from relevant ethics review committees (ERC). We will obtain specific ERC approval and consent from participants for any research involving collection and storage of human material. Data collected will be pseudo anonymised but remain linked to participants using a unique identity number.

The IRD has existing guidelines which have been published,[30], and contain the existing policies and guidelines on access to and sharing data/material. It addresses specific concerns when involving human participants and potential use of human tissue and data collection, documentation, storage and access. These guidelines will be revised and updated accordingly. In addition, in developing the governance structure we will also incorporate the Council for International Organizations of Medical Sciences guidelines for future research: more specifically guideline 12; a governance structure in place to ensure the appropriate use of stored data, which is particularly relevant to the proposed work will be considered.

Consent and information provision

For peer review only - http://bmjopen.bmj.com/site/about/guidelines.xhtml

Consenting to participate in a cohort study is a long-term commitment, often an uncertain one when specific use of data is not known in advance. Including children and minors in such a project requires parental consent but raises challenges as children grow into mature minors and adults. The EEG will give careful thought to how ongoing consent, assent, and re-consenting will be undertaken as children develop into autonomous persons who can engage in, and make decisions about, research participation. Potential participants will be contacted for consent for register inclusion, but database registration does not mandate research participation; appropriate consenting processes will be developed to ensure optimal balance between respect for participants' autonomy and overburdening with individual participation requests. Participants will be informed that registration is entirely voluntary, and that there is no obligation for them to take part. Incentives or payments will not be provided for registering in the twin database.

We will consult the EEG on how best to contact and obtain assent/consent from twins and their parents. Information leaflets (which have received ethical approval) will be provided to all potential participants. The leaflets will emphasise the database registration does not involve mandatory participation in research. And that future research will need separate ethics clearance from the country of origin and Sri Lanka.

Data storage, sharing, access confidentiality and anonymity

Existing IRD institutional policies will be followed to ensure secure data storage and usage to guarantee the obligations of confidentiality and anonymity towards participants. IRD will have ownership of the data collected in future research projects. We will only share anonymised data with academics; however, they will not have direct access to the IRD twin data bases. Personal data of participants will be stored in a confidential, password protected databases accessible by only the IRD team. Any documents that contain non-anonymized data will be stored in line with UK Data Protection Act (1998). The data sharing policy based on existing institutional policies will be agreed at the outset.

Consent forms will be kept in a separate location to the research data used for analysis so that no linkage can occur. All datasets and databases will be stored on a secure, password protected computer in IRD's secured data room. Backups of this data will be done regularly and stored along with any hard copy information in a locked filling cabinet of the data room.

Public involvement

The pioneering work in PPIE which is a relatively novel concept in Sri Lanka and South Asia will provide a unique opportunity to benefit LMIC and HIC as well through reciprocal learning. Evidence from the UK demonstrates that the quality of research is better, and the likelihood of successful recruitment and implementation of the findings is improved when patients and the public are involved in research,[31].

The IRD in collaboration with the Sri Lankan Medical Association and Keele University, UK held a one-day pre-congress workshop at the 131st Anniversary International Medical Congress of the Sri Lanka Medical Association. Resource persons from IRD, Keele University and other guest lectures from Sri Lanka who are experts in PPIE shared their experience and knowledge with 72 workshop participants. This workshop was pre-planned and funded through MRC, UK grant funding obtained for this study.

We aim to establish a culture of PPIE in Sri Lanka, working with both academic colleagues and lay members; in this instance specifically with twins and their parents. Recruiting lay members to a PPIE group to oversee the work described and work with academics to plan and develop future research questions. Training and culturally adapted materials will enhance their engagement. Engaging more professionals/celebrities (parents of twins or twins themselves) in PPIE activities will increase awareness of twin specific issues from a sociological perspective

For peer review only - http://bmjopen.bmj.com/site/about/guidelines.xhtml

Dissemination plan

Engagement, communication and dissemination of research and related activities beyond academia are a priority at the IRD. Since its inception, the IRD worked closely with academic partners, policymakers, the public and the electronic/print media. We will promote twin research through electronic media and print, especially using our own trilingual research and science magazine “Gaveshana”. Gaveshana is published quarterly by the IRD and approved by the Ministry of Education for distribution to schools. Scientific publications in high impact international journals will allow the sharing of knowledge through a wider network. The initiative and the strategy to work beyond twin methods will be widely publicised to attract a wider research community.

TARGETED OUTCOMES OF THE PROJECT AT THE END OF TWO YEAR

Outputs:

1. A sampling frame (the register) of new-born/child/adolescent twins and mothers pregnant with twins with consent to invite for future research.

2. Identification of a core set of potential data variables which are of sufficient quality for research and are feasible to extract from routine records on children’s health and education.

3. Development of key research proposals, taking into consideration research gaps in child and adolescent mental health research.

4. Revision of the existing IRD ethics and governance guidelines.

5. PPIE related wok which will include a twin gathering event organized to initiate the process of developing networks and engaging twins in twin research.

DISCUSSSION

This unique register will increase the volume of high-quality research and generate a wealth of longitudinal data on mental health and other disciplines in a South Asian LMIC. Phenotypic, biological, and anthropometric data, physical measures and social data will allow the studying of health and wellbeing throughout the life course. It will also take cross cultural research into a global context and will provide a better understanding of the mechanisms and dimensions that underlie vulnerability and resilience to mental illness, as well as the personal, social and economic impact of mental illness as a consequence of a wider ranging external drives and risk factors. And popularise twin research methods, provide capacity enhancement for junior academics and advances on ethics and PPIE in Sri Lanka and within the region.

This initiative provides capacity building in ethics for LMIC and UK researchers, especially on issues related to cutting edge collaborative research in LMIC. Capacity building benefit specifically on ethics will include enhanced training and knowledge on vulnerable groups (minors, those with mental illness), proxy consent, and international collaborations. Academics in other disciplines will benefit in this endeavour as well. This will be two-fold: firstly, research beyond mental health, and secondly beyond twin research.: the collection of a wealth of longitudinal phenotypic, biological, anthropometric and physical measures as well as economic, social and psychological data on families with twins. It also enables pioneering education research. The initiative has implications for foetal medicine because twins are associated with prematurity, low birth weight, greater intra-uterine growth retardation, more birth defects, birth complications and neonatal mortality.

Social value arising from the project is through the potential impact on many domains. Potential societal impact will come from highlighting the additional economic, social, and psychological impact of being a twin. Thereby highlighting twins as a unique societal stratum .and creating strong awareness among public and policy planners on the above issues.

For peer review only - http://bmjopen.bmj.com/site/about/guidelines.xhtml

Since there is a constellation of exposures that put adolescents at risk of neurodevelopmental and mental health conditions and many of which may be simultaneous outcomes of other exposures, research should, measure multiple factors,[13]. Therefore, the proposed work will offer a significant opportunity for future LMIC research in diverse disciplines.

A focus on Sri Lankan twins may limit the generalisability of some findings to other populations. The reason is because of the varying impact of environment between HIC and LMIC. Heritability is population-specific and due to genetic differences between populations all ethnic groups, analyses need to be performed in all ethnic groups to understand a trait at a world-wide level. Being the first child and adolescent twin registry in Sri-Lanka will allow to answer questions that could not be studied before Inhabitants of LMIC are often under-represented in genetic studies and therefore the studies arising from this data base will add significant value to the scares research in LMIC,[32].

Acknowledgments

We would like to acknowledge the other members of the **SEARCH Group**: Prof Aasim Ahmad (The Aga Khan University, Pakistan), Dr Achala Jayathilake (Post Graduate Institute of Medicine, Sri Lanka), Prof Ajith Nagahawatte (University of Ruhuna, Sri Lanka), Dr. Anant Bhan (Bioethics and Global Health, India), Dr. Chandima Wickramatilake (University of Ruhuna, Sri Lanka), Dr. Gominda Ponnamperuma (University of Colombo, Sri Lanka), Dr. Lavan Selvarathnam (Ministry of Health, Sri Lanka), Prof Preethi Udagama (University of Colombo, Sri Lanka), Dr. Sorcha Uí Chonnachtaigh, Dr. Steven Blackburn, Dr. Tom Shepherd, and Dr. Toby Helliwell (from (Keele University, UK). Dr. Sudath Samaraweera (Ministry of Health, Sri Lanka), Dr. Thamasi Makuloluwa (General Sir John Kotelawala Defence University, Sri Lanka), Dr. Yatan Balhara (All India Institute of Medical Sciences, Delhi), and Ms Ruwini Cooray (Institute for Research & Development).

We would like to acknowledge Dr. Jayanthi Gunesekara (National Institute of Education), Mrs. Jayani Tillakaratne (Ministry of Education), Mr. Saman Indrajith (The Island Newspaper, Sri Lanka). We also highly appreciate the ongoing support from Dr. Nethanjalee Mapitigama, Dr Kaushalya Kasthuriarachchi, and Dr. Hiranya Jayawickrama of the Family Health Bureau, and Dr. Ruwan Wijayamuni, Chief Medical Officer, Colombo Municipal Council. We would also like to thank higher officials of the Ministry of Health and the Ministry of Education.

We gratefully acknowledge members of the Institute for Research & Development, Dr. Irena Zwierska and Ms. Rebecca Parker at Keele University who have contributed immensely to the preparation and execution of the project.

Funding

This study is supported by a one year pump priming grant awarded by the Medical Research Council, UK (Grant number: MC_PC_MR/R018448/1). AS currently receives salary support in kind from the Midland Partnership Foundation NHS Trust, UK.

Authors' contributions

All authors contributed to the drafting of original funding application which is the basis of this protocol paper. Athula Sumathipala was responsible for the initial draft of the overall concept for the application and the paper, Fruhling Rijsdijk, Richard Saffery, Emily Simonoff, Jeffrey Craig, and Helena M S Zavos were mainly responsible for twin design, Buddhika Fernando Jonathan Ives and Athula Sumathipala were mainly responsible for ethics and governance component, Nihal Abeysinghe Nick Glozier, Asiri Hewamalage, Kelvin Jordan, Christian Mallen, Omar Rahman, Shamza Zafar, Rita Yusuf, Kaushalya Jayaweera and Lasith Dissanayake were mainly responsible for epidemiology and statistics components, Sunil De Alwis, Krysia Dziedzic, and Godwin Kodituwakku were mainly responsible for PPIE component, Alka Saxena, for genetic component, Lasith Dissanayake and Alina Andras contributed to obtaining ethical clearance. Kaush Jayaweera, Lasith Dissanayake and Athula Sumathipala contributed at all stages of planning and writing/editing the article based on the original

For peer review only

funding application. As this is a protocol paper there was no acquisition of data or analysis and interpretation of data. All authors contributed to different drafts of the paper and finally approved the manuscript for submission.

Competing interests

The authors declare that they have no competing interests.

References:

1 Murray CJL, Vos T, Lozano R, *et al.* Disability-adjusted life years (DALYs) for 291 diseases and injuries in 21 regions, 1990–2010: a systematic analysis for the Global Burden of Disease Study 2010. *Lancet* 2012;**380**:2197–223. doi:10.1016/S0140-6736(12)61689-4

2 Patel V, Flisher AJ, Hetrick S, *et al.* Mental health of young people: a global public-health challenge. Lancet. 2007;**369**:1302–13. doi:10.1016/S0140-6736(07)60368-7

3 World Health Organization. Atlas: Child and Adolescent Mental Health Resources: Global Concerns, Implications for the Future. 2005. doi:10.3109/08039487509097356

4 Veena SR, Gale CR, Krishnaveni G V, *et al.* Association between maternal nutritional status in pregnancy and offspring cognitive function during childhood and adolescence; a systematic review. *BMC Pregnancy Childbirth* 2016;**16**:220. doi:10.1186/s12884-016-1011-z

5 Jenkins TA. Perinatal complications and schizophrenia: Involvement of the immune system. Front. Neurosci. 2013. doi:10.3389/fnins.2013.00110

6 Perkonigg A, Kessler RC, Storz S, *et al.* Traumatic events and post-traumatic stress disorder in the community: prevalence,risk factors and comorbidity. *Acta Psychiatr Scand* 2000;**101**:46–59. doi:10.1034/j.1600-0447.2000.101001046.x

7 Georgieff MK. Nutrition and the developing brain: Nutrient priorities and measurement. *Am J Clin Nutr* 2007;**85**:614–20. doi:85/2/614S [pii]

8 Forsyth JK, Ellman LM, Tanskanen A, *et al.* Genetic risk for schizophrenia, obstetric complications, and adolescent school outcome: Evidence for gene-environment interaction. *Schizophr Bull* 2013;**39**:1067–76. doi:10.1093/schbul/sbs098

9 Green B, Friedman M, de Jong J, *et al. Trauma Interventions in War and Peace: Prevention, Practice, and Policy*. 2004.

10 Betancourt TS, Khan KT. The mental health of children affected by armed conflict: Protective processes and pathways to resilience. *Int Rev Psychiatry* 2008;**20**:317–28. doi:10.1080/09540260802090363

11 Giaconia RM, Reinherz HZ, Hauf AC, *et al.* Comorbidity of substance use and post-traumatic stress disorders in a community sample of adolescents. *Am J Orthopsychiatry* 2000;**70**:253–62. doi:10.1037/h0087634

12 Sharan P, Gallo C, Gureje O, *et al.* Mental health research priorities in low- and middle-income countries of Africa, Asia, Latin America and the Caribbean. *Br J Psychiatry* 2009;**195**:354–63. doi:10.1192/bjp.bp.108.050187

13 Davidson LL, Grigorenko EL, Boivin MJ, *et al.* A focus on adolescence to reduce neurological, mental health and substance-use disability. *Nature* 2015;**527**:S161–6. doi:10.1038/nature16030

For peer review only - http://bmjopen.bmj.com/site/about/guidelines.xhtml

14 Jayaweera K, Aschan L, Pannala G, *et al.* The Colombo Twin and Singleton Follow-up Study: a population based twin study of psychiatric disorders and metabolic syndrome in Sri Lanka. *BMC Public Health* 2018;**18**:145. doi:10.1186/s12889-017-4992-2

15 Baxter AJ, Patton G, Scott KM, *et al.* Global Epidemiology of Mental Disorders: What Are We Missing? *PLoS One* 2013;**8**:e65514. doi:10.1371/journal.pone.0065514

16 WHO. Community genetic services: report of a WHO consultation on community genetics in low- and middle-income countries. 2010. doi:10.1159/000080789

17 Plomin R, Haworth CM a. Genetics and Intervention research. *Perspect Psychol Sci* 2010;**5**:557–63. doi:10.1177/1745691610383513

18 Eley TC, Hudson JL, Creswell C, *et al.* Therapygenetics: the 5HTTLPR and response to psychological therapy. *Mol Psychiatry* 2012;**17**:236–7. doi:10.1038/mp.2011.132

19 Sumathipala A, Yelland L, Green D, *et al.* Twins as participants in randomised controlled trials: a review of published literature. *Twin Res Hum Genet*

20 Kendler KS, Baker JH. Genetic influences on measures of the environment: A systematic review. Psychol. Med. 2007;**37**:615–26. doi:10.1017/S0033291706009524

21 Scarr S, McCartney K. How People Make Their Own Environments: A Theory of Genotype - Environment Effects. *Child Dev* 1983;**54**:424. doi:10.2307/1129703

22 Gjerde LC, Czajkowski N, Roysamb E, *et al.* A longitudinal, population-based twin study of avoidant and obsessive-compulsive personality disorder traits from early to middle adulthood. *Psychol Med* 2015;**45**:3539–48. doi:10.1017/S0033291715001440

23 World Health Organization. Regional Office for South-East Asia. WHO country cooperation strategy: 2018--2023, Sri Lanka. 2018.

24 Department of Census and Statistics Sri Lanka. Census of Population and Housing 2012. 2012.

25 Sumathipala Athula, Dhanuja M, Wijesundara H. Challenges of Research in Rural Mental Health in Sri Lanka. In: Chaturvedi S, ed. *Mental Health and Illness Worldwide*. Springer, Singapore 2019. doi:10.1007/978-981-10-0751-4_30-1

26 Siribaddana SH, Siriwardane WD, Hewage SN, *et al.* Update from Sri Lankan Twin Registry: establishment of a population-based twin register and ongoing project on common mental disorders, alcohol abuse and suicidal ideations. *Twin Res Hum Genet* 2006;**9**:868–74. doi:10.1375/183242706779462679

27 Census of Population and Housing 2012 Department of Census & Statistics and Ministry of Policy Planning and Economic Affairs. 2015.

28 Hoekstra C, Zhao ZZ, Lambalk CB, *et al.* Dizygotic twinning. *Hum Reprod Update* 2008;**14**:37–47. doi:10.1093/humupd/dmm036

29 Siribaddana SH, Ball HA, Hewage SN, *et al.* Colombo Twin and Singleton Study (CoTASS): a description of a population based twin study of mental disorders in Sri Lanka. *BMC Psychiatry* 2008;**8**:49. doi:10.1186/1471-244X-8-49

30 Sumathipala A, Siribaddana S. *Research Ethics from a Developing World Perspective*. Vijitha Yapa Publications 2003.

31 Mockford C, Staniszewska S, Griffiths F, *et al.* The impact of patient and public involvement on UK NHS health care: A systematic review. Int. J. Qual. Heal. Care. 2012;**24**:28–38. doi:10.1093/intqhc/mzr066

32 Polderman TJC, Benyamin B, De Leeuw CA, *et al.* Meta-analysis of the heritability of human traits based on fifty years of twin studies. *Nat Genet* 2015;**47**:702–9. doi:10.1038/ng.3285

For peer review only

For peer review only
